# A draft physical map of a D-genome cotton species (*Gossypium raimondii*)

**DOI:** 10.1186/1471-2164-11-395

**Published:** 2010-06-22

**Authors:** Lifeng Lin, Gary J Pierce, John E Bowers, James C Estill, Rosana O Compton, Lisa K Rainville, Changsoo Kim, Cornelia Lemke, Junkang Rong, Haibao Tang, Xiyin Wang, Michele Braidotti, Amy H Chen, Kristen Chicola, Kristi Collura, Ethan Epps, Wolfgang Golser, Corrinne Grover, Jennifer Ingles, Santhosh Karunakaran, Dave Kudrna, Jaime Olive, Nabila Tabassum, Eareana Um, Marina Wissotski, Yeisoo Yu, Andrea Zuccolo, Mehboob ur Rahman, Daniel G Peterson, Rod A Wing, Jonathan F Wendel, Andrew H Paterson

**Affiliations:** 1 Plant Genome Mapping Laboratory, University of Georgia, Athens, GA, 30605, USA; 2 Department of Ecology, Evolution, & Organismal Biology, Iowa State University, Ames, IA 50011, USA; 3 Arizona Genomics Institute, School of Plant Sciences and BIO5 Institute for Collaborative Research, University of Arizona, Tucson, AZ 85721, USA; 4 National Institute for Biotechnology & Genetic Engineering (NIBGE), Faisalabad, Pakistan; 5 Life Sciences & Biotechnology Institute, Mississippi State University, Mississippi State, MS 39762 USA; 6 Department of Plant Biology, University of Georgia, Athens, GA, 30602, USA; 7 School of Agriculture and Food Sciences, Zhejiang Forestry University, Lin'an, Hangzhou, Zhejiang, 311300, China; 8 Department of Plant and Microbiology, College of Natural Resources, University of California, Berkeley, CA, USA

## Abstract

**Background:**

Genetically anchored physical maps of large eukaryotic genomes have proven useful both for their intrinsic merit and as an adjunct to genome sequencing. Cultivated tetraploid cottons, *Gossypium hirsutum *and *G. barbadense*, share a common ancestor formed by a merger of the A and D genomes about 1-2 million years ago. Toward the long-term goal of characterizing the spectrum of diversity among cotton genomes, the worldwide cotton community has prioritized the D genome progenitor *Gossypium raimondii *for complete sequencing.

**Results:**

A whole genome physical map of *G. raimondii*, the putative D genome ancestral species of tetraploid cottons was assembled, integrating genetically-anchored overgo hybridization probes, agarose based fingerprints and 'high information content fingerprinting' (HICF). A total of 13,662 BAC-end sequences and 2,828 DNA probes were used in genetically anchoring 1585 contigs to a cotton consensus genetic map, and 370 and 438 contigs, respectively to *Arabidopsis thaliana *(AT) and *Vitis vinifera *(VV) whole genome sequences.

**Conclusion:**

Several lines of evidence suggest that the *G. raimondii *genome is comprised of two qualitatively different components. Much of the gene rich component is aligned to the *Arabidopsis *and *Vitis vinifera *genomes and shows promise for utilizing translational genomic approaches in understanding this important genome and its resident genes. The integrated genetic-physical map is of value both in assembling and validating a planned reference sequence.

## Background

The *Gossypium *(cotton) genus, composed of 50 species among which four provide the major raw material for one of the world's largest industries (textiles), has a large impact on our economy and everyday life. Diploid cottons are classified into 8 genome types, denoted A-G and K, based on chromosome pairing relationships [1]. All diploid cotton species are believed to have shared a common ancestor about 5-10 million years ago [1]. The cotton genome types diverged into genome groups that vary in haploid genome size from 2500 Mb in the K genome, to less than 900 Mb in the D genome [2,3], while retaining common chromosome number (n = 13) and largely-collinear gene order [4-7]. The tetraploid cotton genome is thought to have formed by an allopolyploidy event about 1-2 million years ago, involving species similar to the modern New World D genome species *G. raimondii *(GR) [8] or *G. gossypioides *(GG) [9] and the Old World A genome species *G. herbaceum *(GH).

There exist at least a dozen published genetic maps for various *Gossypium *crosses, most involving members of the superior-fiber-quality *G. barbadense *species crossed with high-yielding *G. hirsutum*. These maps collectively include > 5,000 public DNA markers (~3,300 RFLP, 700 AFLP, >2,000 SSR, and 100 SNP). Many thousands of additional SSRs have been described [10], but only a subset of these have been mapped [4,11-13]. The most detailed sequence tagged site (STS)-based map, and a source of probes for many of the other maps, are reference genetic maps for diploid (D) and tetraploid (AtDt, where Dt refers to the D-subgenome found in tetraploid cottons (to distinguish it from the genome of D-diploid cottons). Likewise, At refers to the A-subgenome of tetraploid cottons.) *Gossypium *genomes that include respectively, 2584 loci at 1.72 cM (~600 kb) intervals based on 2007 probes (AtDt); and 1014 loci at 1.42 cM (~600 kb) intervals detected by 809 probes (D) [4,14]. A high degree of collinearity among the respective genome types permitted inference of the gene order of a hypothetical common ancestor of the At, Dt, and D genomes for 3016 loci identified by 2337 probes, spanning 2324.7 cM [14]. Additional maps that are particularly marker-rich and/or have been widely used as reference maps for QTL studies have been developed from three additional interspecific crosses [11-13]. Other important resources include aneuploid substitution stocks that were derived from tetraploid genotypes TM-1 (*G. hirsutum*) × 3-79 (*G. barbadense*) [15] and TM-1 × *G. tomentosum *[16]. Together, monosomics and telosomics have been used to assign 20 of the 26 cotton linkage groups to chromosomes, and the remaining six linkage groups were assigned to chromosomes by translocation and fluorescence *in situ *hybridization mapping [17].

Cotton genetic maps have been employed in identification of diagnostic DNA markers for a wide range of traits related to fiber yield and quality [18-46]; drought tolerance [46-48]; and resistance to diseases [49-54], and pests [55-59]. Interest in hybrid cottons in some countries has drawn attention to a nuclear restorer of cytoplasmic male sterility [60-64]. Morphological features such as the pubescence that is characteristic of *G. hirsutum *[65-68], leaf morphology [69-72] and color [73], and unique features such as nectarilessness [34,69,74] have also received attention. The value of cotton seed has led to interest in mapping variation in seed physical characteristics and nutritional value [75]. Meta-analysis of multiple QTL mapping experiments by alignment to a common reference map has begun to reveal the genomic organization of trait variation [76]. Although members of the D genome clade do not make spinnable fiber, genetic mapping has shown that the majority of fiber QTLs mapped in tetraploid cotton fall on D genome (*G. raimondii*-derived) chromosomes, suggesting that the D genome has been crucial to the evolution of the higher fiber quality and yield of cultivated tetraploid cottons [76].

Toward the long-term goal of characterizing the spectrum of diversity among the 8 *Gossypium *genome types and three polyploid clades, the worldwide cotton community has prioritized the D-genome species *Gossypium raimondii *for complete sequencing [77,78]. *Gossypium raimondii *is a diploid with a ~880 Mb genome [3], the smallest genome in the *Gossypium *genus at ~60% of the size of the diploid A genome and 40% of the tetraploids. It is largely inbreeding, and a largely-homozygous genotype has been used in both a reference genetic map [4] and for a BAC library (herein). DNA renaturation kinetics shows that 30-32% of the *G. raimondii *genome contains repetitive DNA, with a kinetic complexity of 1.6 × 10^6 ^bp and an average iteration frequency of ~120 copies per haploid genome [79]. This has been subdivided into a highly-repetitive component of about 5% of the genome, composed of elements in 10,000 or more copies; and a middle-repetitive component accounting for 27% of the genome [80]. A random sampling of 0.04% of the tetraploid cotton genome, enough to sample repetitive element families that occur in 2500 or more copies, revealed only 4 D-genome-derived elements ranging in estimated copy number up to about 15,000, versus dozens of A-genome-derived repeats at much higher copy numbers [81]. Pilot sequencing studies (X. Wang, D. Rokhsar, A.H. Paterson, unpubl.) show that most D-genome repetitive DNA families are sufficiently heterogeneous to be compatible with a whole-genome shotgun approach.

Genetically anchored physical maps of large eukaryotic genomes have proven useful both for their intrinsic merit and as an adjunct to genome sequencing. In species where no whole-genome sequence is yet available, a physical map is a useful tool in a wide range of activities including comparative genomics and gene cloning. Physical mapping also provides a method of genome assembly independent of a sequence, and is useful in contributing to and/or validating whole-genome shotgun sequences [82]. For BAC-based sequencing of a genome, a physical map is a prerequisite. Recent study of chromosomes 12 and 26 of upland cotton (*Gossypium hirsutum*) [83] suggests that physical mapping of polyploid cotton may be complicated by homoeologous genome fragments.

As an important step toward its genome-wide characterization, we describe here a genetically anchored, BAC-based physical map for *G. raimondii*. By incorporating thousands of DNA markers, the physical map is tightly integrated with the rich history of cotton molecular genetics research described above, and expedites a host of studies of *Gossypium *biology and evolution. Moreover, comparison of the physical map to the sequences of *Arabidopsis thaliana *and *Vitis vinifera *shows promise for utilizing translational genomic approaches in better understanding the structure, function, and evolution of this important genome and its resident genes.

## Results

### BAC library

The *Gossypium raimondii *BAC library used in physical mapping consists of 92,160 clones. Pulsed-field gel electrophoresis-based examination of 448 *Not*I digested clones indicates a mean insert size of 100 kb. Of note, there was little variation in insert size among clones (standard error of mean = 0.76). Three of the 448 interpretable *Not*I-digested clones (i.e., 0.67%) appear to be false positives. Likewise, three of the 4032 BAC end sequences generated from the library exhibit homology to chloroplast DNA (0.07%) indicating that the methods employed in constructing the library [84] were successful in keeping chloroplast contamination low. Collectively, the library affords 10× coverage of the *G*. *raimondii *genome.

### Agarose-based fingerprints and HICF

**Figure 1:**
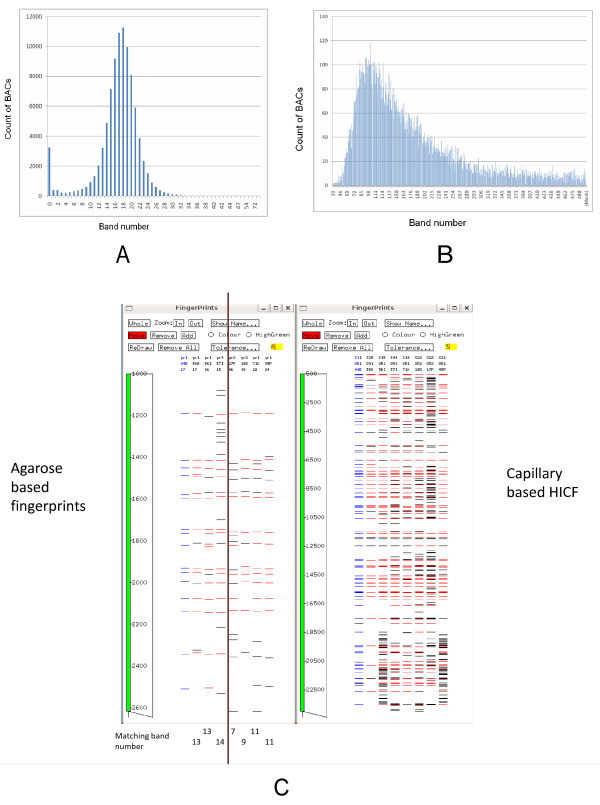
**Comparison of band number distribution between agarose-based fingerprints and HICF**. A: agarose-based fingerprints; B: HICF; C: an example of two agarose FPC contig joined in HICF. Red bands are matching bands to the highlighted (in blue) BAC. Counts of matching bands to the BAC are listed below each lane. The four BACs on the right were not assembled into the same contig.

Two different types of fingerprints were employed in this study: a preliminary assembly used agarose-based fingerprinting and an improved assembly resulted from re-fingerprinting a subset of BACs using HICF. The entire 92,160-clone GR BAC library was fingerprinted using slight modification of established agarose-based fingerprinting methods [85]. Preliminary assembly formed 9,290 contigs and 26,716 singletons at a tolerance value of 8 and cutoff value of 1e-10. The average agarose-based fingerprint band number of individual BACs was 17.4. Band number distribution across the library is shown in Figure 1A. A total of 3266 BACs failed to produce usable fingerprints.

Two terminal BACs from each end of the largest 4608 agarose contigs (four BACs per contig, totaling 18,432 BACs) from the preliminary assembly were fingerprinted using HICF. The average HICF band number per BAC was initially 203.6. HICF batches with extremely high or low band numbers (approximately top or bottom 5%) were re-fingerprinted. The average band number dropped to 178. These 18,432 BACs formed 3508 contigs and 2570 singletons. The final band number distribution is shown in Figure 1B.

### Overgo hybridizations

Thousands of probes were applied to the GR library using a multiplex hybridization scheme (see Methods). A total of 2828 probes from *Arabidopsis *genes, cotton ESTs, and genetic markers showed hybridization signal attributable to one or more BACs by this approach. On average, each probe hit 17.3 BACs. A total of 46 probes hit more than 100 BACs and are considered highly repetitive. To minimize false associations, probes with >50 hits were not used in the contig assembly process, and probes with >30 hits were not used in the contig anchoring process (detailed later). Thus, 2658 probes (with <50 hits) were integrated into the assembly using the CpM table in FPC: stringency (cutoff value) was relaxed by 2, 3, or 4 denary (ten-fold) intervals when 1, 2 and 3+ common markers were found between two BACs.

### Integrated assembly

Since agarose-based fingerprinting and HICF use different sets of restriction enzymes, a different band-calling scheme, and have different error rates and band size tolerances, data from these two different methods cannot be merged directly. Further, while we targeted HICF to contig-terminal BACs, it would be imprudent to declare a join in the agarose assembly whenever HICF suggests a merge of contig-terminal BACs, overlooking potential false joins in HICF. To circumvent this, if two agarose contig-terminal BACs were suggested to be joined by HICF, we lowered the cutoff value for joining agarose contig-terminal BACs by two denary intervals, e. g. when the overall cutoff was set to 1e-12, we would accept an overlap at the cutoff at 1e-10 if the two BACs were found in the same HICF contig. The agarose assembly was thus reassembled, only forming a merged contig if it was supported by both data types (see Methods), and integrating 2658 hybridization markers based on 2828 overgos.

Collectively, the agarose fingerprints, targeted HICF fingerprints, and overgo hybridization data joined a total of 67,343 BACs into 4208 contigs, leaving 21,551 singletons. Based on the average insert size estimate of 100 kb, and an estimated genome size of 880 Mbp [3], the 67,343 BACs in contigs provide ~7.7× coverage of the GR genome. The majority of contigs (61.5%) contain between 3 and 25 BACs. The distribution of BAC numbers per contig is shown in additional file 1.

Singletons differed in several ways from BACs in contigs. The average agarose-based fingerprint band number was 13.4 for singletons, versus 17.9 for BACs in contigs. A total of 9476 (44% of) singletons contained less than 12 bands. This could reflect either shorter length of singleton BACs, or the presence of tandem repeats that produce fingerprint bands that comigrate, reducing the scoreable band number and perhaps contributing to failure of some BACs to form contigs (see more discussion of band numbers below). A total of 1904 overgo probes hit singleton BACs, among which 364 overgos were repetitive and 1540 were low copy (having <30 hits total). Compared to the probes that hit BACs in contigs (376 repetitive and 2129 low copy), singletons show some enrichment in repetitive DNA content. A total of 585 singletons were identified as possible cross-well contaminations.

### Anchoring contigs to the cotton consensus map

After filtering out 381 (of 2828) repetitive overgo probes that hit more than 30 BACs in the GR library, and 357 BACs (out of 34,713 BACs with at least one marker hit) with more than 8 markers hybridized as suspected hybridization artifacts, the remaining probes and BACs produced 40,152 BAC-probe pairs. A total of 7772 of these were produced by BACs that were not in contigs (singletons); 5946 of the markers on contigs were "weak anchors" produced by a single BAC-probe pair for the contig. Weak anchors were not used in aligning the contigs onto the genetic map. The remaining 26,434 BAC-probe pairs derive from 1920 probes, and were distributed in 2154 contigs.

**Figure 2:**
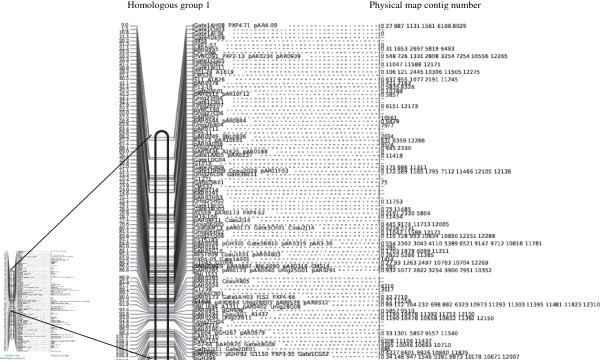
**Aligned physical map contigs along the consensus map**. Homologous Group 1 of the integrated genetic-physical map, drawn using data from Rong *et al. *[14].

A 'consensus' cotton genetic map built from the At, Dt and D genome genetic maps contains 13 homologous groups made up of 3016 loci based on 2337 unique sequence tags [14]. Among these, 2109 have probes designed (961 RFLP probes and 1744 overgos, 596 have both, most of the remainder could not be sequenced). After filtering out probes with >30 hits in the library, 1468 loci on the consensus map have anchored 1586 contigs. (Table 1, Figure 2, S1). On average, each marker anchored 2.42 contigs.

**Table 1:** Distribution of anchored contigs on consensus chromosomes.

Homologous Group	number of loci	contig anchoring markers	anchored contigs	average # of contigs per marker
1	245	119	288	2.42
2	194	101	249	2.47
3	149	74	169	2.28
4	208	95	278	2.93
5	246	103	292	2.83
6	235	122	354	2.90
7	290	129	345	2.67
8	247	111	282	2.54
9	382	184	527	2.86
10	164	98	265	2.70
11	227	117	314	2.68
12	187	105	280	2.67
13	242	110	330	3.00

Grand Total	3016 (2234 unique)	1468 (1144 unique)	3973 (1585 unique)	2.42

### Aligning contigs to *Arabidopsis thaliana *and *Vitis vinifera *whole-genome sequences

A total of 8064 BACs selected from the ends of the largest 2016 contigs from the preliminary assembly were used for paired-end sequencing. The resulting 13,662 high-quality sequences, along with the 1920 low copy probes (after filtering described above), were used in comparing the GR contigs to *Arabidopsis thaliana *(AT) and *Vitis vinifera *(VV) chromosomes.

BAC end-sequences (BES) and the source sequences of the hybridization probes were aligned to the AT and VV whole-genome sequences using BLASTn. A total of 2607 sequences (1370 BES and 1237 overgo source sequences) had between 1 and 9 BLAST hits in the AT genome, and 2968 sequences (1557 BES and 1411 overgo source sequences) have between 1 and 9 hits in the VV genome. (Sequences with >10 hits were excluded as repetitive.)

**Figure 3:**
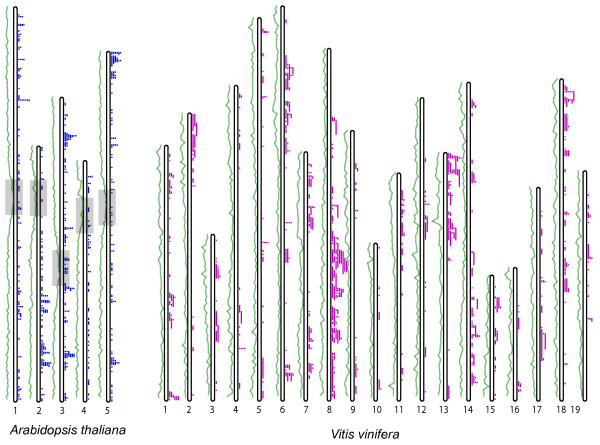
**Aligned GR contigs along *Arabidopsis *and *Vitis *chromosomes**. Blue and purple bars next to the chromosomes show the GR contigs. Green lines to the left of each chromosome indicate gene-density of the target genomes. The length of the bar represents the physical distance between anchoring markers on the target genomes. Putative centromeric regions on *Arabidopsis *chromosomes are marked out in rectangles. Centromeric regions on *Vitis *chromosomes cannot be determined.

A total of 370 contigs were aligned to *Arabidopsis *chromosomes, 438 to *Vitis *chromosomes, and 242 to both (Table 2, Figure 3). All 566 that aligned contained 64 CB units (consensus band units, the number of total non-overlapping bands in a contig) per contig on average, about 50% larger than the overall average contig size (42 CB units). Based on an estimated size of 4097 bps per band (average of all band sizes from all BACs fingerprinted), these contigs cover a minimum of 13% (contigs anchored on VV) and 11% (contigs anchored on AT) of the GR genome, noting that band numbers somewhat underestimate contig sizes because both very large and very small bands are excluded from bandcalling. A second estimate of coverage of the target genomes by aligned contigs was obtained by adding up the distances between anchor marker BLAST matches and excluding overlaps. This suggests that 27.7% of the *Arabidopsis *genome and 22.8% of the *Vitis *genome is covered by aligned GR contigs. Some contigs have significant association with two or more positions on a target genome. The distributions of contigs along AT and VV chromosomes are shown in Figure 3. Contigs are more likely to be anchored to two or more locations in AT than VV (159 or 43% of contigs anchor to multiple AT locations versus 111 or 25.4% of contigs anchored to VV), consistent with the fact that the *Arabidopsis *lineage has experienced two more whole-genome duplication (WGD) events than grape [86].

**Table 2:** Number of anchored contigs on each chromosome of *Arabidopsis thaliana *(AT) and *Vitis vinifera *(VV) genomes

AT chr	number of contigs anchored
Chr1	132
Chr2	126
Chr3	168
Chr4	72
Chr5	152

Total	650 (370 unique)

	

VV chr	number of contigs anchored

1	36
2	21
3	19
4	21
5	28
6	53
7	41
8	96
9	14
10	8
11	21
12	18
13	59
14	45
15	17
16	11
17	17
18	57
19	18

Total	600 (438 unique)

The GR contigs anchored on VV are not evenly distributed across the chromosomes, but rather are clustered in several regions/chromosome arms that tend to have higher than average gene densities. Gene density distribution across the *Vitis *genome was extracted by counting the number of genes in 200 kb bins along the chromosomes. Gene density is largely uniform across the *Arabidopsis *chromosomes except for the centromeric regions; while in the *Vitis *genome, we observed greater heterogeneity of gene density. The regions on which we were able to anchor GR contigs (Figure 3) had an average of 20 genes per 200 kb window, versus an average of 14.8 for the remainder of the genome. Among the 30% of VV 'windows' with highest gene density, 37.9% were covered by GR contigs; versus 22.8% of the genome as a whole.

### Nature of repetitive probes

A total of 46 probes are classified as highly-repetitive (with >100 BAC hits). These came from several sources: 28 were derived from cotton EST sequences (COV), 3 were derived from genes that are low-copy in *Arabidopsis *(AOG), and 15 were derived from cotton RFLP probes used in genetic mapping (see additional file 2 for the complete sources of these probes). Six of the highly repetitive cotton overgo sequences were found to be located within known repetitive elements using Repbase http://www.girinst.org/repbase/. The overgo with the most hits (COV1526, which hits 1593 BACs) is in a helitron. The remaining five were from two *hAT*-like DNA transposons, one EnSpm element, one ERV/ERV2 element and one *Gypsy *element. Four of the 15 highly repetitive PCR-based probe sequences contain repetitive elements. The three *Arabidopsis *genes from which highly repetitive overgos were designed (At5g10360, At2g30740 and AtGRF2) showed no known repetitive elements in their sequences, which might indicate cotton lineage-specific gene multiplications. Given that Repbase does not include a comprehensive set of cotton repetitive sequences (due to lack of a complete *Gossypium *genome), it is likely that the remaining highly repetitive overgos that did not match repetitive sequences from Repbase may reveal cotton elements not previously known to be repetitive.

### Low-copy and repetitive DNA loci were concentrated in different regions of the genome

A total of 3060 contigs contain BACs to which one or more probes hybridized. Probes were classified as low copy (<30 hits total), moderately repetitive (31-97 hits), or highly repetitive (>100 hits). Accordingly, contigs were tentatively classified as repetitive or low-copy based on the ratio of repetitive probes versus low-copy probes hybridized to each contig. A total of 761 contigs contain only repetitive probes, and 1262 contigs contains mostly (>60%) low copy probes. Because a large number of the probes are designed from cotton EST sequences or *Arabidopsis *genes, contigs with relatively more hybridization anchors from low copy probes and relatively fewer from repetitive probes are likely to be gene rich. The 1262 low-copy probe enriched contigs contain 1786 of the 2300 non-repetitive probes. The majority of the low-copy probe enriched contigs (901 out of 1262, or 71.4%) are anchored to the cotton consensus map (additional file 3). By comparison, only 37.7% (1586 out of 4208) of contigs overall could be anchored to the consensus map.

Repetitive contigs are slightly shorter than contigs enriched in low copy probes (average 38.32 CB units versus 44.35 CB units). This could be caused by co-migrating fragments produced by the repetitive sequences that reduce the total number of bands.

### Low-copy probe enriched contigs appear to be largely euchromatic

Among the 438 contigs that showed microsynteny to VV chromosomes, 218 are enriched in low-copy probes and only 14 are repetitive probe enriched. Similarly, among the 370 contigs that showed microsynteny to AT chromosomes, 166 were enriched in low copy probes, and only 17 are repeat-enriched. This is consistent with our prior findings in other taxa that microsynteny tends to be preserved in gene-rich euchromatic regions but not in repeat-rich heterochromatic regions [87]. We tacitly assume that the 761 repeat-enriched contigs are likely to be largely from heterochromatic regions of the genome and the 1262 low-copy sequence-enriched contigs are likely to be from euchromatic regions of the genome. The 1262 low-copy contigs can be estimated to cover 26% of the genome based on the estimated genome size of 880 M and average band size of 4097 bp. Based on the 68% of the genome estimated to be low-copy by renaturation kinetics[79], these contigs may cover about 38.2% of the low-copy DNA. Contigs aligned to VV and AT genomes contains 1150 (50%) and 954 (41.5%) of all non-repetitive probes. The low copy probes that were unable to align were partly due to the limitation of BLAST in searching across distant related species and the variation in gene density in VV genome.

### Consequences of ancient duplications in the *Arabidopsis thaliana *genome

**Figure 4:**
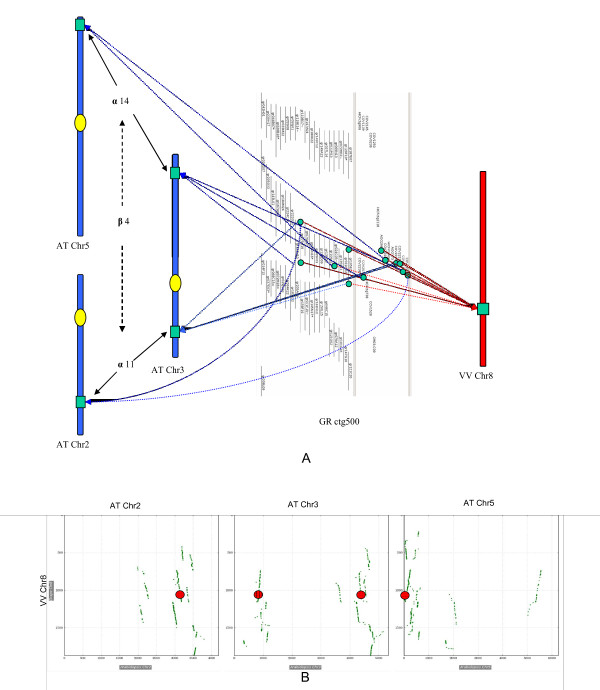
**Alignment of contig 500 to the genome sequences**. A. The contig is mapped to four regions in *Arabidopsis*, which are paralogs produced by the α and β duplications after the cotton-*Arabidopsis *divergence. The contigs are only anchored to a single *Vitis *chromosomal location. B. dot plot generated by MCscan on Plant Genome Duplication Database, showing conserved syntenic blocks between *Vitis *chr.8 and *Arabidopsis *chromosomes. The region corresponding to GR ctg 500 is marked by red circles.

To illustrate the alignment of GR contigs on the AT and VV genomes, ctg500 was used as an example. The contig is anchored to a single VV chromosomal location at about 14.7 Mb on chr8, and to four different locations on the AT genome, at 15 Mb on chr2, 2.7 Mb on chr3, 20 Mb on chr3 and 0.1 Mb on chr5 respectively (Figure 4A). These four AT regions were previously shown to be paralogous segments created by two rounds of whole-genome duplication [88]. The chromosomal region in *Vitis *has also been identified using MCScan [89], to have conserved collinearity with the four AT regions (Figure 4B). Ctg500 is anchored on cotton consensus homologous group 2, at around 67 cM. Based on cotton DNA markers, this region has shown evidence of homology to *Arabidopsis *α11 and α14 groups [14].

### The *G. raimondii *chloroplast

By aligning to the chloroplast DNA sequence of upland cotton (*Gossypium hirsutum*) using BLAST, BAC-end sequences and probes likely to be of chloroplast origin were identified. Ctg11556 is identified as a chloroplast contig. The contig contains 20 BACs, 10 of which are "buried" in FPC, meaning they have nearly identical band patterns as other BACs in the contig, indicating very high similarity among these BACs. COV1960, an overgo probe designed from the sequence of the chloroplast psaJ gene, hits 17 of the 20 BACs in the contig. Three BACs from the contig have end sequences, all of which correspond to the published *G. hirsutum *chloroplast sequence (additional file 4). Based on low-coverage genomic sequencing with some targeted finishing, a D-genome chloroplast sequence has been assembled and is being described (M. Rahman, A. H. Paterson, in prep.)

### GO analysis of BES and shotgun sequences

The 13,662 BES were analyzed using Blast2Go to obtain a distribution of functional gene groups. A total of 9042 did not have significant hits using BLASTx against NCBI nr database, 3234 of the sequences are annotated, and 963 were mapped, but not annotated. No significant differences were observed between the GO distribution of BES and random shotgun sequences except that more genes involved in localization processes were represented in the random shotgun sequences. (see additional files 5 and 6).

## Discussion

The first whole-genome physical map of a cotton species has provided new tools and information, and foreshadows the picture of cotton genome organization prior to the completion of the D-genome sequencing currently in progress. The genetically anchored contigs are potentially helpful in efforts such as gene cloning and local sequence analysis, by providing region-specific BAC resources for marker development and chromosome walking. On a genomic level, comparative analysis between cotton, *Arabidopsis*, and *Vitis *genomes illustrates the potential for translational genomics across these species, and several regions with an unusually high degree of conserved collinearity may be interesting for further research.

Several lines of evidence herein suggest that the *G. raimondii *genome is comprised of two qualitatively different components, specifically one that is gene-rich and recombinogenic with gene repertoire and order that is still recognizably similar to those in members of other angiosperm families (*Vitis, Arabidopsis*), and another that is repeat-rich and recombinationally-recalcitrant with relatively few genes that are highly rearranged relative to their homologs in other taxa. This general picture of cotton genome organization is similar to the picture that emerged from comparison of two monocot genomes, rice and sorghum [82,87].

Curiously, we were able to anchor more contigs on the *Vitis *genome despite the closer relationship of cotton to *Arabidopsis*. This difference is attributable in part to differences in anchoring parameters (see Methods), but also reflects the relatively slow evolution of *Vitis *[89], and highlights the value of the *Vitis *genome as a botanical model for cross-taxon comparative genomic studies.

The present genome assembly remains somewhat fragmented and may be further improved as more information and new technology emerges. Adding more genetically anchored STS to the BACs, as well as mapping of more BAC-derived sequences will permit anchoring of more contigs to their corresponding chromosomal locations.

### Further improvements of the genetic-physical map

While contigs covering ~40% of the genome have been genetically anchored, a higher density of genetic markers may permit anchoring of many more contigs. Some genetically mapped probes hit only singleton BACs and were not incorporated into the physical map in the interest of minimizing false positives. Nearly 1000 probes that hybridized to GR BACs are from sequences that have not yet been genetically mapped, so are not useful in linking the genetic and physical maps. Designing new overgo probes from mapped sequence-tagged sites can be done recursively as more densely populated genetic maps become available. Conversely, new SSR markers can be developed from BES and put onto the genetic map, which would help anchor more contigs and help confirm the position of those already anchored.

### Probes targeted at specific regions of interest

Marker density on the physical map reflects efforts to enrich specific genomic regions containing genes of interest for DNA markers. Most prominent are probes aimed at the *Li1 *(Ligon lintless-1) and *Li2 *(Ligon lintless-2) genes of cotton. About 300 overgo probes were designed from genetic markers and EST reads that showed relationship to the regions of these genes. This enrichment created "hotspots" where more GR contigs could be aligned to both *Arabidopsis *and *Vitis *(Figure 3). In the AT genome, there is an excess of anchored GR contigs near the bottom of chromosome 2, the upper and lower parts of chromosome 3, and the tip of chromosome 5. These four regions were identified in earlier studies [88] to have been produced by two rounds of whole-genome duplication, all belonging to the consensus group β4. Likewise, the regions near the top of VV chromosome 13 and bottom of chromosome 8 anchor a higher than average number of GR contigs.

A closer look at these "hotspots" revealed that the majority of the contigs anchored here contain probes from the *Li1 *and *Li2 *regions. There are 114 contigs anchored in the AT regions described above, 94 (82.5%) of which contains *Li1 *and/or *Li2 *probes. In 87 out of these 94 cases, the *Li *probes provided one or more anchor point(s) in the microsynteny detection. In grape, a total of 134 contigs fell into the most densely anchored regions on grape chromosome 6, 8 and 13; 111 (82.8%) of these contigs contain *Li1 *or *Li2 *probes of which 92 provided one or more anchor point(s) in microsynteny detection. Compared to the whole-genome average of 23% (970) contigs that contains *Li *probes, these regions shows a significant enrichment in *Li *contigs and the ability to align to the AT and VV genomes.

This illustrates the potential use of the contig assembly in cross genome comparisons, and that the power to detect synteny and align contigs across genomes can be greatly increased by targeted enrichment of specific regions for hybridization probes.

### The grape genome as a model

Aligning physical map contigs with sequenced genomes has proven informative in several ways [87,90]. Comparative mapping data and BES alignments to the human genome helped in assigning bovine physical map contigs to their respective chromosomes [90]. The pattern of sorghum physical map contigs along rice chromosomes has given empirical evidence that gene rearrangement is generally deleterious [87]. Cross-species synteny information has also enabled us to make better use of the sequenced genome data on other genomes.

For cotton, *Arabidopsis *is the most closely-related genome for which a sequence is published as of this writing. The rapid evolution of, and two additional WGD events in, the *Arabidopsis *lineage may reduce our ability to align these respective genomes. The *Vitis *genome, on the other hand, evolves relatively slowly [89] and has experienced no WGD events apart from the hexaploidy (γ) event that is likely to be shared by all dicots [86,91]. The grape genome might prove to be more useful than that of *Arabidopsis *in comparative genomics across distantly related species.

One disadvantage of using the grape genome as a model for cotton lies in its relative low gene density compared to the *Arabidopsis *genome. Unlike sorghum and rice, where the euchromatic regions have a similar gene density in both genomes [82,87], gene density is at least twice as high in *Arabidopsis *as in *Vitis*. Gene density across the currently assembled grape pseudomolecules fluctuates from about 20 to 25 genes per 200 kb in higher gene density regions to 10 to 15 genes per 200 kb in lower gene density regions. Similar analysis showed that gene density is uniformly 50 to 60 genes per 200 kb across the *Arabidopsis *genome, except for the centromeric regions and a few low density points with 30 to 40 genes per 200 kb. This lower gene density in *Vitis *reduces our ability to anchor cotton contigs, and look for synteny using contig information. Here, we were able to anchor cotton contigs onto most of the gene dense regions of the *Vitis *genome, but large parts of the low-gene-density chromosomal regions are not covered.

### Using the genetic-physical map in gene cloning

Map-based cloning has always been a long and tedious process. The genetic-physical map provides a shortcut by which contigs spanning a target gene region can be readily identified through flanking markers. Markers immediately upstream and downstream of a target gene can be used to identify neighboring anchored contigs, and sequencing of BACs within the contig(s) could provide candidate genes warranting further study (Table 3). In efforts to characterize a gene involved in cotton fiber development, we were able to identify a contig that anchors to the genetic region of interest using this method, and design new genetic markers very close to the gene (unpublished data).

**Table 3:** contigs identified to flank or contain gene sequences of interest.

Gene name		Closest Contigs
**Cotton fiber genes**	**Closest Genetic Markers**	

*Li2*	A1552, Gate4BC11	ctg1749, ctg2409
*Li1*	Gate4CA09, Coau1J04	ctg478, ctg8796, ctg10964, ctg11826
*N1/Fbl*	Gafb28I12, pAR0244, Gafb29C08	ctg11567, ctg3941, ctg5857, ctg 497, ctg1153, ctg1422, ctg1955, ctg2775, ctg3754, ctg8492, ctg11632, ctg93, ctg4489, ctg12050, ctg10883, ctg7872

Other genes and gene families	Probes	

CesA	COV2311, COV2312, COV1269, COV1270	ctg10763, ctg10561, ctg10858, ctg12310, ctg228
GTPB	COV2309, COV2310	ctg11814, ctg483, ctg669
AdhA	COV1992, COV1993, COV1265, COV1266	ctg10740, ctg11096, ctg11218, ctg11323, ctg12283
AdhC	COV1267	ctg1376
AdhD	COV1924, COV1925	ctg1376, ctg159

*Arabidopsis *trichome genes		

TUA6	-	ctg1653, ctg3177
TTG2	COV1942	ctg937
ACT2	COV1933	ctg908
GL2	-	ctg601
FRA1	COV1932	ctg6359, ctg1785, ctg59
FRA2	COV1940	ctg1009, ctg11648
GL3	COV1945	ctg686, ctg2610
GL1	COV1950	ctg9085, ctg11801
TRY	COV1936	ctg471, ctg3808
SPIKE1	COV1937	ctg10915, ctg157, ctg627

In our efforts to anchor the contigs through probe hybridization, overgo probes were also designed from specific gene sequences (Table 3). Some probes were designed from specific gene families, e.g. COV2311, COV2312, COV1269 and COV1270 were designed from cotton *CesA *gene. BACs and contigs that contain these sequences were identified, which provide materials for study of these gene families. Probes were also designed to identify contigs that include *Arabidopsis *trichome gene homologs. Table 3 shows a list of probes and contigs that is directly applicable to the study of specific genes.

The value of the physical map for positional cloning would be further enhanced by anchoring more contigs onto the genetic maps efficiently and accurately. We have provided a framework on which more than 1500 contigs has been aligned. In genomic regions that are of high priority to specific research efforts (positional cloning, etc), many unanchored contigs might be tentatively merged into the anchored contigs, given a lower stringency or higher tolerance for questionable clones, then seeking additional corroborative data such as additional BAC ends, hybridization anchors, or targeted genetic mapping of hybridizing elements. For regions where no contigs have been anchored yet, a simple probing of the library using flanking genetic markers should be able to help build a local genetic-physical map. Contigs upstream and downstream of a target contig can be identified by manually searching for similar contigs at a lower cutoff, and rebuilding the contigs for the region of interest.

Microsynteny information permits one to utilize new ways of developing genetic markers targeted to a region of interest [92] that may be of high value in translating functional information from botanical models to cotton. The contigs aligned to the AT and VV genomes cover about 1/4 of these respective genomes, primarily in regions that are likely to be gene-rich. Earlier research has identified some *Arabidopsis *genes with well-defined roles in trichome (including root hair) development that approximately correspond to the locations of cotton fiber QTLs. Some of these genes are in regions which showed conserved organization with the GR physical map contigs. e.g. an α-tubulin gene (TUA6) is found in a region spanned by contig1653 and contig3177; the TTG2 gene, which is involved in trichome pattern formation [93], is in a region spanned by contig937; the ACT2 gene, which involves in trichome morphogenesis [94], is in a region spanned by contig908; the GL2 gene is spanned by contig601. These anchorings may provide a good starting point to search for candidate genes and QTLs with similar functions in cotton fiber development, and help elucidate the similarities and differences in trichome formation in different tissues.

### Average band number is crucial in agarose based fingerprinting

The use of both agarose based and HICF methods in this physical map assembly gave us the opportunity to directly compare these two methods that have been widely used in genome projects. Using only the agarose based fingerprints, we obtained a large number of small contigs. To test if this is caused by the low band number, we estimated the expected contig number under our conditions. When cutoff = 1e-12, the minimum matching band number required to call an overlap between two clones is 12 in our study. With a tolerance value of 7 and cutoff of 1e-12, the expected contig number in the assembly would be over 9000 if the average band number per clone is 17. In other words, our agarose based contigging yielded the expected result.

The expected contig number drops rapidly with increased average band number. From the Lander-Waterman formula (seem Methods), if the average band number is increased to 20, the expected contig number would be about 5000. With an average band number of 30, one would expect only about 400 contigs. This should be an underestimation because we are not considering physical gaps and under-represented parts of the genome in the BAC libraries, but nevertheless, shows how critical band numbers are to an agarose-based fingerprinting project. BACs with fewer than 8 bands offer too little information to form statistically-supported contigs, even with identical band patterns.

Our success with using HICF in a targeted manner to improve the physical map stems from much higher band numbers. HICF merged contig-end BAC pairs had average agarose band numbers that are not significantly different from the overall band number (18.02 vs. 18.15 in all BACs in contigs). The reason why they failed to join is due to the high percentage of matching bands needed to call an overlap. FPC was unable to call an overlap even if 11 bands were matching (Figure 1C).

## Conclusion

The first genetically anchored whole-genome physical map of a cotton species was built through integration of agarose-based fingerprinting and 'high information content fingerprinting' (HICF). Integrating genetically-anchored overgo hybridization probes and BAC end sequences permitted many physical map contigs to be aligned to a consensus cotton genetic map as well as *Arabidopsis *and *Vitis *genome sequences. The cotton genome appears to include two qualitatively different components, specifically one that is gene-rich and recombinogenic with gene repertoire and order still recognizably similar to those in members of other angiosperm families, and another that is repeat-rich and recombinationally-recalcitrant with relatively few genes that are highly rearranged relative to those of other angiosperms. While *Vitis *appears to be a more informative comparator regarding cotton genome organization, translational genomics from *Arabidopsis *offers singular benefits in identifying the functions of cotton genes. In summary, the physical map is (a) a link that connects genetic map information with physical sequences; (b) a means of validating/directing whole-genome shotgun sequencing assembly; and (c) a tool providing insight into the genome organization of cotton, in advance of a whole-genome sequence.

## Methods

### BAC library construction

The *Gossypium raimondii *(GR) BAC library was constructed by D.G.P. according to Peterson *et al. *[84]. The library consists of 92,160 individually-archived clones and is available through the Plant Genome Mapping Laboratory http://www.plantgenome.uga.edu. To estimate mean insert size and false positive percentage, two clones were selected from each of the library's 240 384-well plates, and minipreps of these clones were digested with *Not*I and analyzed by pulsed-field gel electrophoresis. Of the 480 digested clones, 448 produced interpretable banding patterns; the remaining 32 were not visible on the gels suggesting that the DNA was lost in the miniprep procedure. Three of the 448 clones appear to be false positives.

### Probe design and hybridization

A total of 2828 sequence-tagged site probes were hybridized to the GR library: 357 were overgos designed from *Arabidopsis *genic sequences (prefixed AOG); 1751 were designed from genetically mapped cotton markers (prefixed COV for cotton overgos, or CM/COAU/PAR for PCR based probes); and 252 from cotton EST sequence reads (prefixed COV). The rest were designed and probed from cotton genes of interest related to multiple projects. Overgo probes [95] were designed and hybridized to the libraries as described [87]. Briefly, source sequences were aligned to all known plant sequences to using BLAST to find conserved domains, and compared to known plant repeats to screen out possible repetitive sequences. The selected sequences were then chopped into 40 bp segments and screened for GC content of between 40% and 60%.

Probes were labeled using P-32 and applied to macroarrays of 18,432 BACs per membrane in a multiplex of 576 probes, using pools of 24 probes per bottle, by rows, columns and diagonals of a 24 × 24 array of probes. Films were manually scored, scores digitized using text-recognition software (ABBYY FINEREADER), and data deconvoluted and stored in the MS Access database system "BACMan".

### Fingerprinting

Agarose based fingerprinting methods were adapted from Marra *et al. *[85]. Plasmids were extracted in batches of 96-well plates and digested using *Hin*dIII. Fragments were separated on a 121-lane 1% agarose TAE gel, with a size standard every 5 lanes. Band migration distances and molecular weights were digitized using IMAGE [96], before importing into FPC (**F**inger**p**rinted **C**ontigs) [97,98].

High information-content fingerprinting (HICF) was adapted from published methods [99]. Plasmids were digested with *EcoRI, BamHI, XbaI, XhoI *and *HhaI*. The ends of restriction fragments were differentially labeled using fluorochrome tagged ddNTPs after the first four enzyme cuts, and the last enzyme further reduced fragment size and produced a blunt end. Fingerprints were generated using an ABI3730xl sequencer and size files generated by GeneMapper v4.0 after processing the chromatograms.

### Cross-well contaminations and chimeric clones in HICF

Cross-well contaminations seem to be a more severe problem in HICF than in agarose-based FPC assembly. In our first HICF assemblies, we encountered a very large contig containing as many as ~50% of all BACs, depending on the assembly stringency. To overcome this issue, 1166 BACs were excluded from the assembly due to suspiciously high band numbers (possible chimeras) or by a newly implemented function in FPC to identify potential cross-well contaminations. The new assembly show no contigs containing >24 BACs.

### Physical map assembly

Agarose-based fingerprints were assembled first by FPC using a cut-off value of 1e-10 and a tolerance value of 8. CpM (contigs plus markers) tables were used to integrate the marker hybridization results: the cut-off value was relaxed to 1e-8, 1e-7 and 1e-6 when two BACs shared one, two and three markers respectively.

After the preliminary assembly, two BACs from each end of the largest 4608 agarose FPC contigs were subjected to HICF. These fingerprints were assembled separately in FPC using a cut-off value of 1e-50 and a tolerance of 3. Overgo hybridization information was not used in HICF assembly. Results from HICF were formatted into a marker file, and fed into the final, integrated assembly in the same manner as probe hybridization results. In this assembly, cutoff was set to 1e-12 and tolerance was set to 7. CpM tables were used in integrating the data. Cut-off values were relaxed to 1e-10, 1e-9 and 1e-8 when two BACs shared one, two and three markers (or HICF contig) respectively.

In each of the three iterations of assembly, the final stringency settings (tolerance and cut-off) were determined by comparing results of different cut-off and tolerance value combinations. For HICF, tolerance values of 2 through 5 and cut-off value of 1e-20 through 1e-50 were tested; for agarose fingerprints, tolerance values of 6 through 9 were and cut-off value of 1e-10 through 1e-12 were tested. Possible cross-well contaminations were identified and rendered as singletons using the built-in function under "search commands" in FPC v 9.3.

### Finalizing the assembly

End-to-end auto-merges were done recursively by lowering the cut-off value one step at a time, from 1e-12 through 1e-6. Singletons were also merged into the assembly recursively using the Keyset-to-FPC function in the FPC program. The CB maps for each contig with 2 or more Q clones were recalculated using a higher stringency cutoff value. Q-contigs were thus split up by FPC into smaller contigs and singletons. This was done recursively by raising the cutoff value by 1 level at a time until each one of the splitted contigs contains no more than 1 Q clone. A tarball containing all data (both agarose-based fingerprints and HICF) is available at http://www.plantgenome.uga.edu/pgml_image_data/.

### Simulation of contig number change with average band number

FPC uses the Sulston score [96] as a cutoff criterion to call overlaps, . This is the probability of finding *j *matching bands in two BACs with *n *bands each. Expected contig numbers were predicted using the Lander-Waterman formula [100], *E(contig#) *= *Ne*^-*LN/G·(1-T/L) *^, where *G *is the genome length (genome size/average band size), *L *is the average band number; *N *is the number of BACs fingerprinted and *T *is the number of bands needed to call an overlap. In our study, the gel length is 5000 bands, the genome size is 880 Mb, and the average band size is 4096 bps (for a 6-cutter).

### BAC-end sequencing

Two BACs from each end of the largest 2016 contigs were end-sequenced by the Arizona Genome Institute using methods as previously described [101].

### Anchoring contigs onto genetic maps

To achieve a maximum number of anchor points, a 13-linkage-group consensus map of cotton, constructed by integration of At, Dt, and D genome genetic maps [14] was used to anchor contigs. Probes that hit only one BAC in a contig were considered possible hybridization artifacts and were not used; probes that hit 30 or more BACs in the GR library were considered repetitive and were also excluded. 482 BACs with 8 or more different probes hybridized to them were excluded as possible contamination artifacts produced in hybridization. Contigs were aligned to the consensus map using the remaining anchor markers.

On average, we had less than one hybridization marker per contig, and the vast majority of contigs had less than three anchor probes. Thus, instead of requiring the contig to have two or more anchor markers from proximal regions on the genetic map to call an anchor, we listed all the contigs anchored by one or more genetic markers alongside the marker's location(s) on the genetic map.

### Aligning contigs to whole-genome sequences

BAC-end sequences (BES) and source sequences of overgo probes were used to BLAST against *Arabidopsis thaliana *and *Vitis vinifera *genome sequences, using a penalty score of -2 (instead of -3 as the default value) and a e value of 1e-5 in BLASTn. The penalty score was changed to fit the sequence divergence among genomes surveyed, so that longer hits with lower similarity (66.7%) can be retained. *Arabidopsis *and *Vitis *genome sequences were downloaded from TAIR ftp://ftp.arabidopsis.org/home/tair/Sequences/whole_chromosomes/ and Genoscope http://www.genoscope.cns.fr/externe/Download/Projets/Projet_ML/data/8X/assembly/goldenpath/unmasked/, respectively.

Sequences with 10 or more BLAST hits in either genome were considered repetitive and excluded from later analysis. Probe hybridization results used the same filters described for anchoring to genetic maps. BAC contigs were then linked to the genomic sequences through the BLAST data in a MS Access database query. The query results were processed by a Python script aligning the contigs to a genomic region of AT or VV when two or more sequences from the same contig hit a genomic region less than 200 kb (against AT) or 1 Mbp (against VV) apart.

## Authors' contributions

AHP, RAW and JFW conceived and designed the experiments; DGP and CL constructed and prepared the BAC library; GJP, ROC and LL generated BAC fingerprints; JCE, JEB, LKR, CK, SK, LL, CG, NT and MR designed and performed the overgo hybridizations; AHC, EE, KLH, JI, JO, EU and SK scored the hybridization results; MB, KC, WG, DK, MW, YY and AZ performed BAC-end sequencing; LL, JEB and HT analyzed the data. Reagents/materials/analysis tools were provided by AHP, JCE, HT, XW and RAW; LL and AHP wrote the manuscript. All authors read and approve the final manuscript.

## Supplementary Material

Additional file 1 **The distribution of contigs sizes of the integrated assembly**. Contig sizes were measured by the number of BACs contained in one contig. The majority of contigs contain between 3 and 25 BACs. Click here for file

Additional file 2 **Sources of the 46 highly repetitive probes**. Probes with >100 BAC hits in the GR library and the locus from which the probes were derived. Click here for file

Additional file 3 **A consensus genetic-physical map of the cotton genome**. The position of the 1585 genetically anchored physical map contigs on the consensus genetic map integrating the At, Dt and D genome genetic maps. Click here for file

Additional file 4 **GR chloroplast contig**. Contig11556 is identified as a chloroplast contig, with BAC-end sequences and an overgo probe aligned to the GH chloroplast sequence. Click here for file

Additional file 5 **Gene ontology analysis result of the *G. raimondii *sequences**. GO classification results generated from 13662 BAC-end sequences and 13661 random shotgun sequences, using Blast2Go at an ontology level of 2. (Details refer to additional file 6). Click here for file

Additional file 6 ***G. raimondii *preliminary gene ontology classification results**. GO classification results generated from 13662 BAC-end sequences and 13661 random shotgun sequences, using Blast2Go at an ontology level of 2. Click here for file
