# Supplementary figures and images for: A draft physical map of a D-genome cotton species (Gossypium raimondii)
Source: BMC Genomics. 2010 Jun 22;11:395. doi: 10.1186/1471-2164-11-395 (PMC2996926; doi:10.1186/1471-2164-11-395)

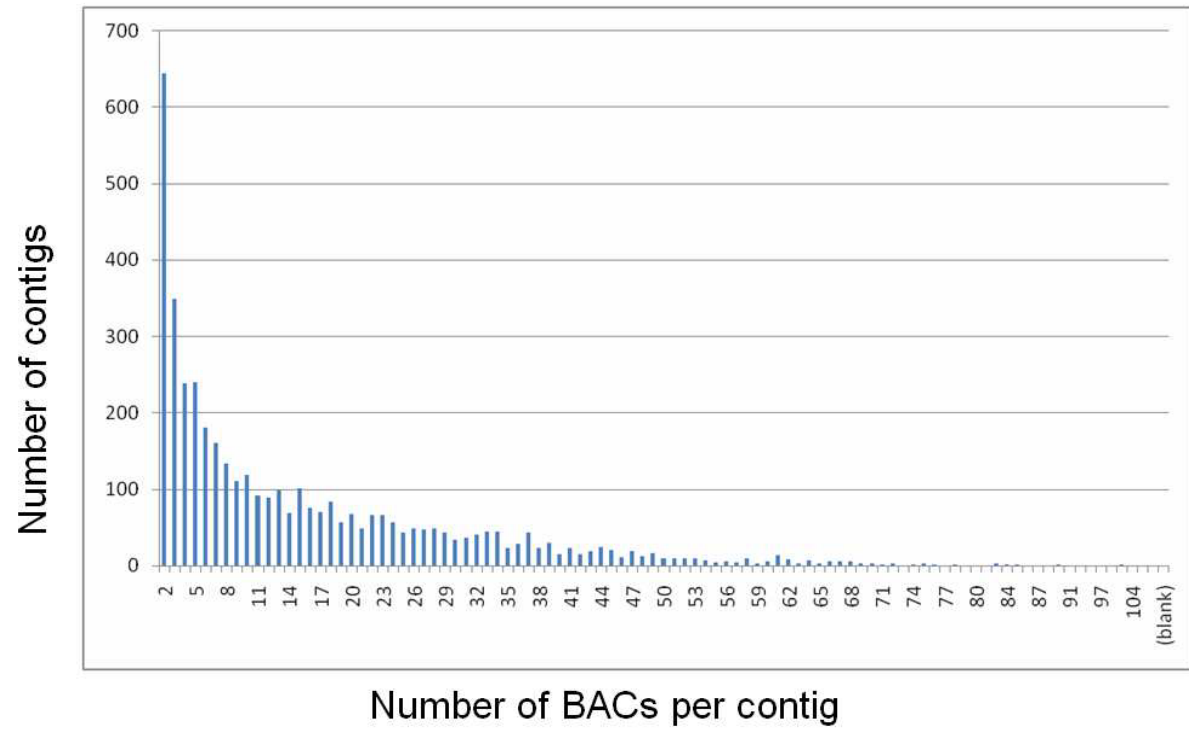

Supplement: Additional file 1 — The distribution of contigs sizes of the integrated assembly. Contig sizes were measured by the number of BACs contained in one contig. The majority of contigs contain between 3 and 25 BACs. [file 1471-2164-11-395-S1.PDF]

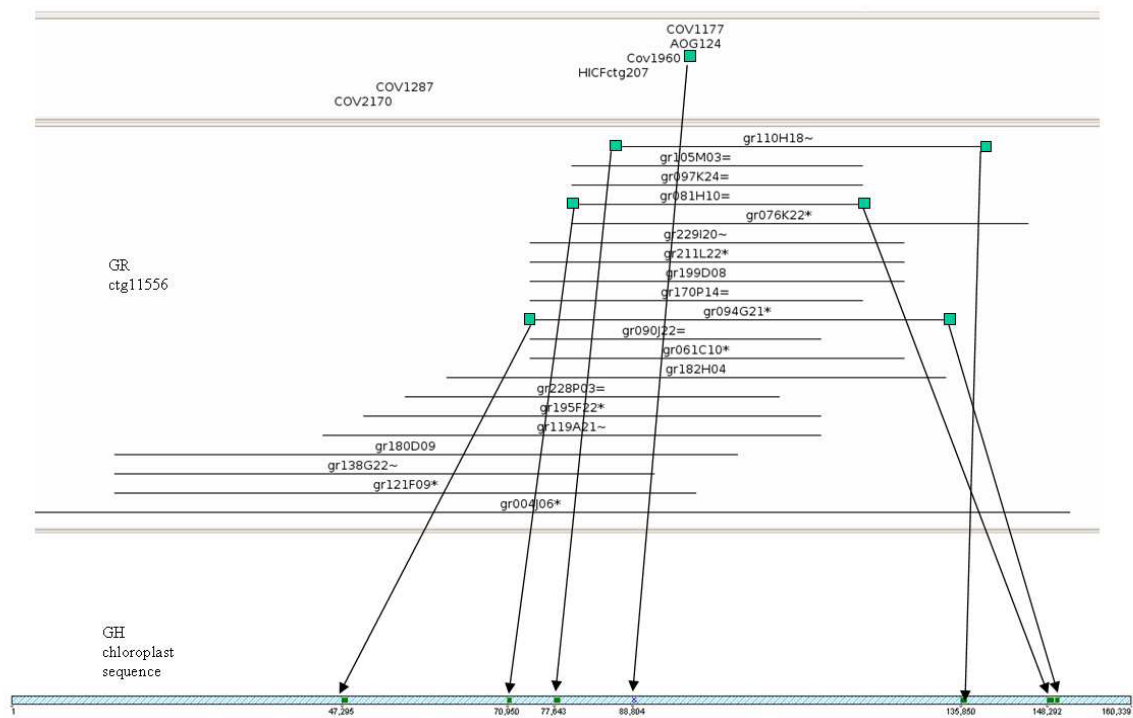

Supplement: Additional file 4 — GR chloroplast contig. Contig11556 is identified as a chloroplast contig, with BAC-end sequences and an overgo probe aligned to the GH chloroplast sequence. [file 1471-2164-11-395-S4.PDF]

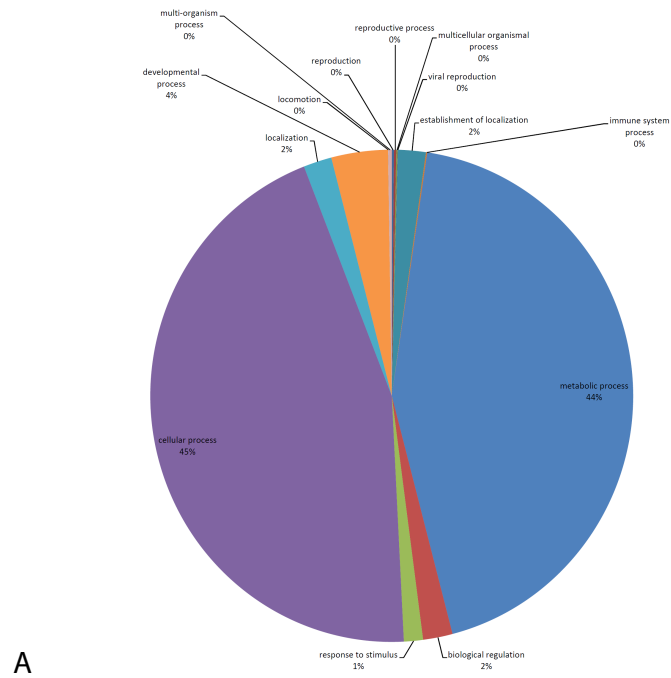

A

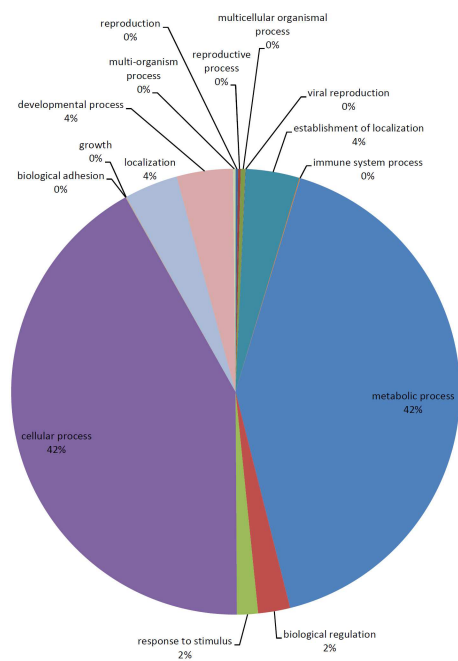

B

Supplement: Additional file 5 — Gene ontology analysis result of the G. raimondii sequences. GO classification results generated from 13662 BAC-end sequences and 13661 random shotgun sequences, using Blast2Go at an ontology level of 2. (Details refer to additional file 6). [file 1471-2164-11-395-S5.PDF]
