# Supplementary material for: A draft physical map of a D-genome cotton species (Gossypium raimondii)
Source: BMC Genomics. 2010 Jun 22;11:395. doi: 10.1186/1471-2164-11-395 (PMC2996926; doi:10.1186/1471-2164-11-395)
Supplement: Additional file 3 — A consensus genetic-physical map of the cotton genome. The position of the 1585 genetically anchored physical map contigs on the consensus genetic map integrating the At, Dt and D genome genetic maps. [file 1471-2164-11-395-S3.PDF]

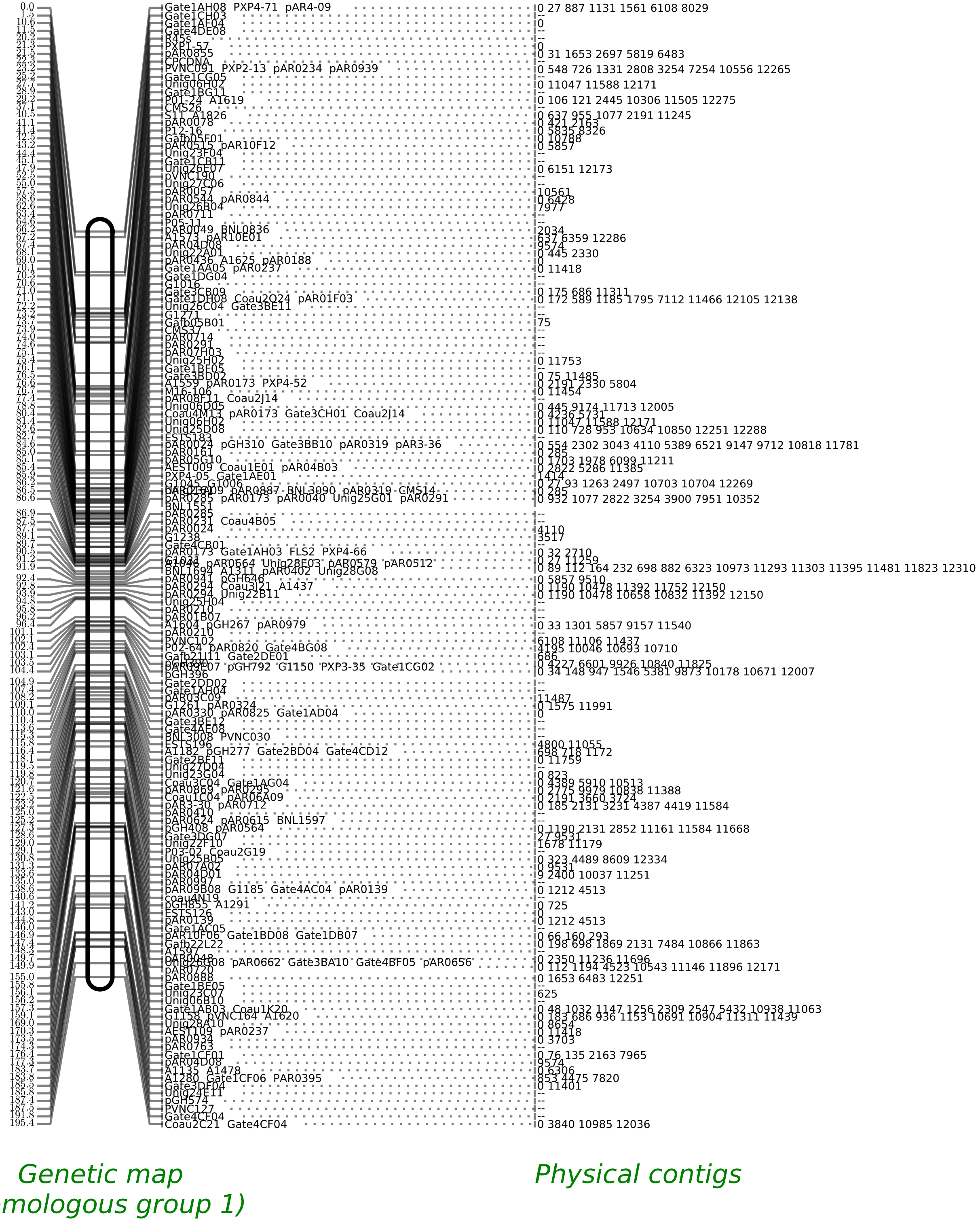
Genetic map
(Homologous group 1)
Physical contigs

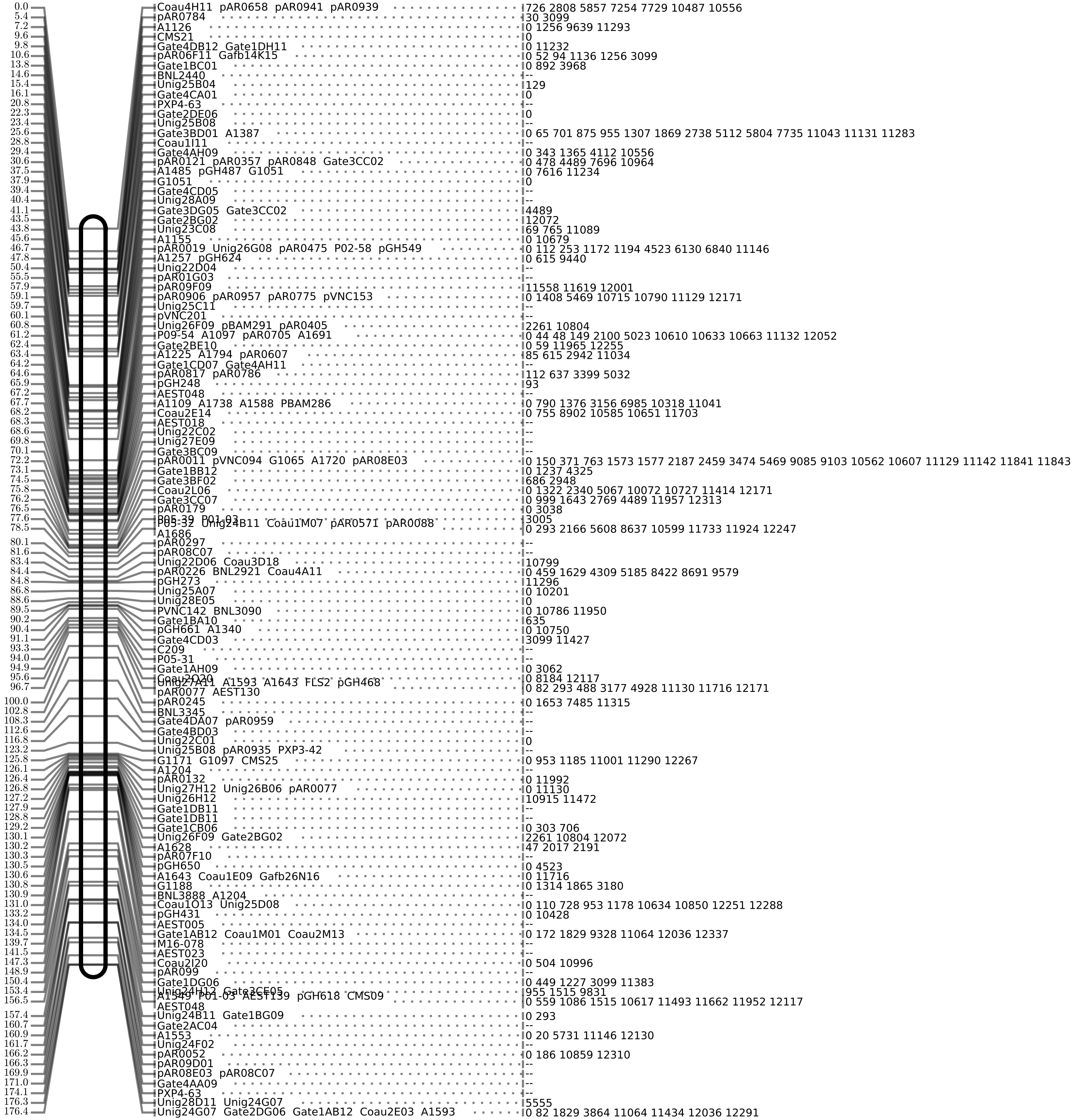

Genetic map (Homologous group 2)

Physical contigs

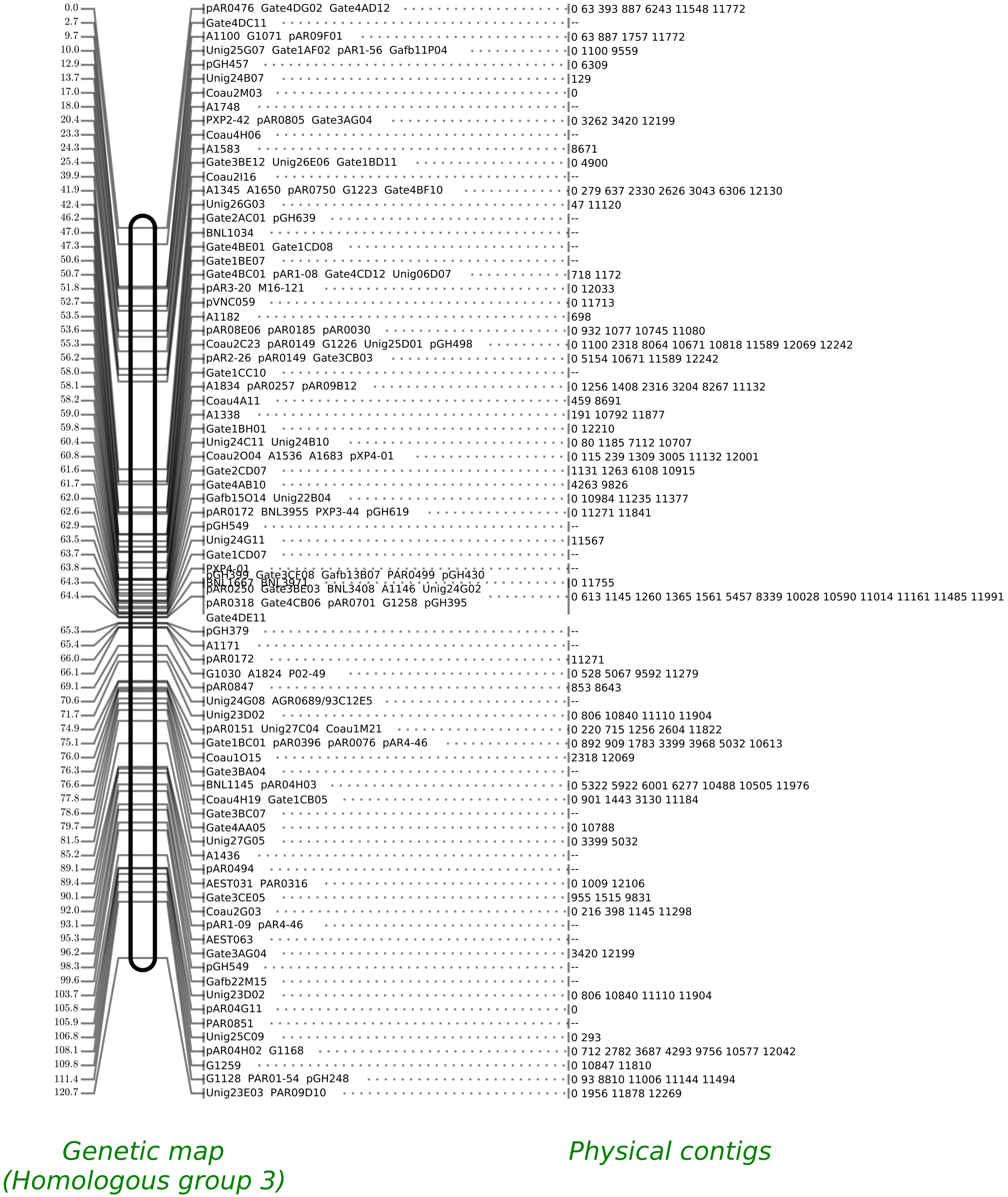

0.0 pAR0476 Gate4DG02 Gate4AD12 0 63 393 887 6243 11548 11772
2.7 Gate4DC11 --
9.7 A1100 G1071 pAR09F01 0 63 887 1757 11772
10.0 Unig25G07 Gate1AF02 pAR1-56 Gafb11P04 0 1100 9559
12.9 pGH457 0 6309
13.7 Unig24B07 129
17.0 Coau2M03 0
18.0 A1748 --
20.4 PXP2-42 pAR0805 Gate3AG04 0 3262 3420 12199
23.3 Coau4H06 --
24.3 A1583 8671
25.4 Gate3BE12 Unig26E06 Gate1BD11 0 4900
39.9 Coau2I16 --
41.9 A1345 A1650 pAR0750 G1223 Gate4BF10 0 279 637 2330 2626 3043 6306 12130
42.4 Unig26G03 47 11120
46.2 Gate2AC01 pGH639 --
47.0 BNL1034 --
47.3 Gate4BE01 Gate1CD08 --
50.6 Gate1BE07 --
50.7 Gate4BC01 pAR1-08 Gate4CD12 Unig06D07 718 1172
51.8 pAR3-20 M16-121 0 12033
52.7 pVNC059 0 11713
53.5 A1182 698
53.6 pAR08E06 pAR0185 pAR0030 0 932 1077 10745 11080
55.3 Coau2C23 pAR0149 G1226 Unig25D01 pGH498 0 1100 2318 8064 10671 10818 11589 12069 12242
56.2 pAR2-26 pAR0149 Gate3CB03 0 5154 10671 11589 12242
58.0 Gate1CC10 --
58.1 A1834 pAR0257 pAR09B12 0 1256 1408 2316 3204 8267 11132
58.2 Coau4A11 459 8691
59.0 A1338 191 10792 11877
59.8 Gate1BH01 0 12210
60.4 Unig24C11 Unig24B10 0 80 1185 7112 10707
60.8 Coau2O04 A1536 A1683 pXP4-01 0 115 239 1309 3005 11132 12001
61.6 Gate2CD07 1131 1263 6108 10915
61.7 Gate4AB10 4263 9826
62.0 Gafb15O14 Unig22B04 0 10984 11235 11377
62.6 pAR0172 BNL3955 PXP3-44 pGH619 0 11271 11841
62.9 pGH549 --
63.5 Unig24G11 11567
63.7 Gate1CD07 --
63.8 PXP4-01 --
64.3 pGH399 Gate3CF08 Gafb13B07 PAR0499 pGH430
BNL1667 BNL3971 0 11755
64.4 pAR0250 Gate3BE03 BNL3408 A1146 Unig24G02
pAR0318 Gate4CB06 pAR0701 G1258 pGH395
Gate4DE11 0 613 1145 1260 1365 1561 5457 8339 10028 10590 11014 11161 11485 11991
65.3 pGH379 --
65.4 A1171 --
66.0 pAR0172 11271
66.1 G1030 A1824 P02-49 0 528 5067 9592 11279
69.1 pAR0847 853 8643
70.6 Unig24G08 AGR0689/93C12E5 --
71.7 Unig23D02 0 806 10840 11110 11904
74.9 pAR0151 Unig27C04 Coau1M21 0 220 715 1256 2604 11822
75.1 Gate1BC01 pAR0396 pAR0076 pAR4-46 0 892 909 1783 3399 3968 5032 10613
76.0 Coau1O15 2318 12069
76.3 Gate3BA04 --
76.6 BNL1145 pAR04H03 0 5322 5922 6001 6277 10488 10505 11976
77.8 Coau4H19 Gate1CB05 0 901 1443 3130 11184
78.6 Gate3BC07 --
79.7 Gate4AA05 0 10788
81.5 Unig27G05 0 3399 5032
85.2 A1436 --
89.1 pAR0494 --
89.4 AEST031 PAR0316 0 1009 12106
90.1 Gate3CE05 955 1515 9831
92.0 Coau2G03 0 216 398 1145 11298
93.1 pAR1-09 pAR4-46 --
95.3 AEST063 --
96.2 Gate3AG04 3420 12199
98.3 pGH549 --
99.6 Gafb22M15 --
103.7 Unig23D02 0 806 10840 11110 11904
105.8 pAR04G11 0
105.9 PAR0851 --
106.8 Unig25C09 0 293
108.1 pAR04H02 G1168 0 712 2782 3687 4293 9756 10577 12042
109.8 G1259 0 10847 11810
111.4 G1128 PAR01-54 pGH248 0 93 8810 11006 11144 11494
120.7 Unig23E03 PAR09D10 0 1956 11878 12269
Genetic map
(Homologous group 3)
Physical contigs

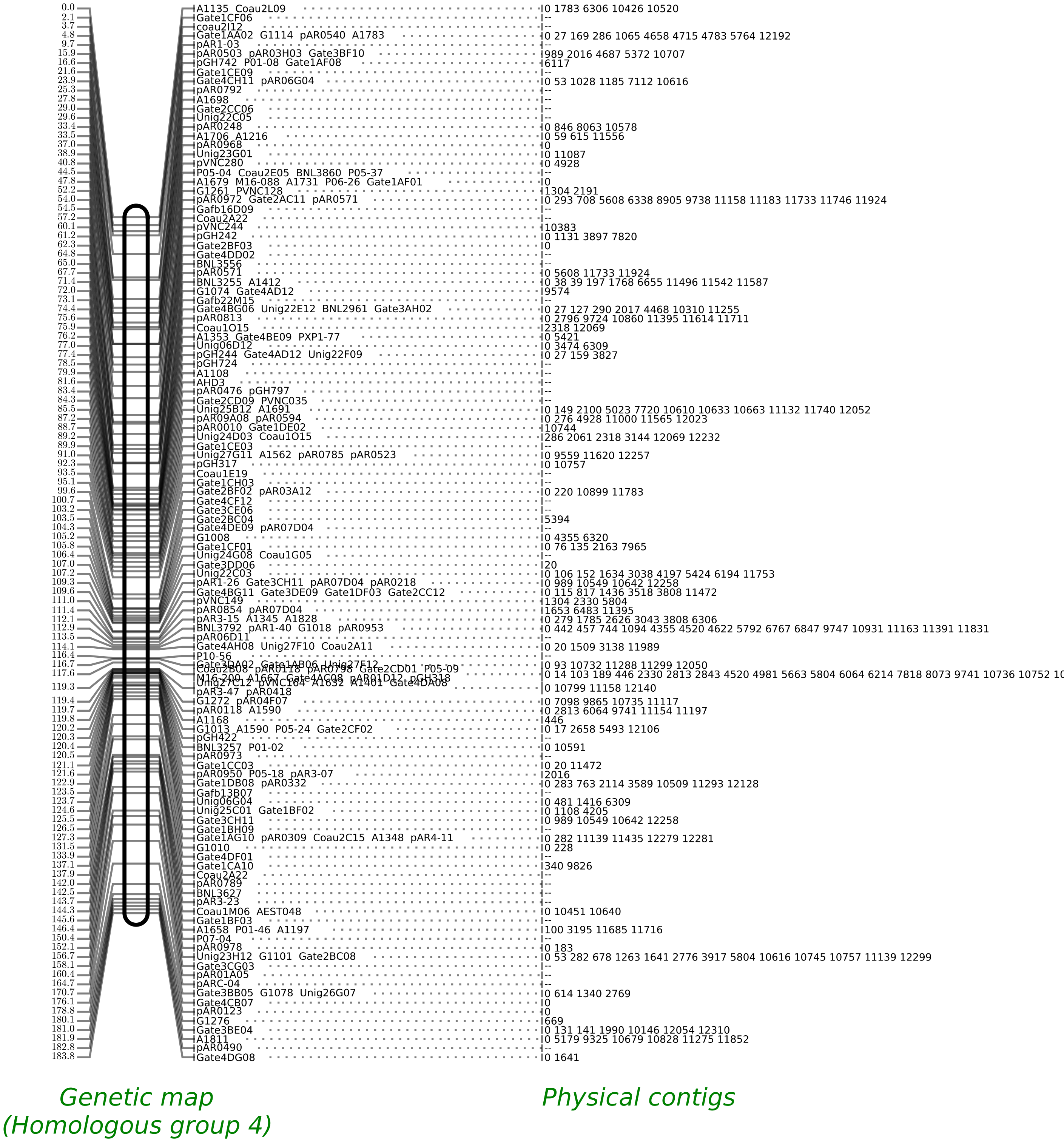
0.0
2.1
3.7
4.8
9.7
15.9
16.6
21.6
23.9
25.3
27.8
29.0
29.6
33.4
33.5
37.0
38.9
40.8
44.5
47.8
52.2
54.0
54.5
57.2
60.1
61.2
62.3
64.8
65.0
67.7
71.4
72.0
73.1
74.4
75.6
75.9
76.2
77.0
77.4
78.5
79.9
81.6
83.4
84.3
85.5
87.2
88.7
89.2
89.9
91.0
92.3
93.5
95.1
99.6
100.7
103.2
103.5
104.3
105.2
105.8
106.4
107.0
107.2
109.3
109.6
111.0
111.4
112.1
112.9
113.5
114.1
116.4
116.7
117.6
119.3
119.4
119.7
119.8
120.2
120.3
120.4
120.5
121.1
121.6
122.9
123.5
123.7
124.6
125.5
126.5
127.3
131.5
133.9
137.1
137.9
142.0
142.5
143.7
144.3
145.6
146.4
150.4
152.1
156.7
158.1
160.4
164.7
170.7
176.1
178.8
180.1
181.0
181.9
182.8
183.8
A1135 Coau2L09 0 1783 6306 10426 10520
Gate1CF06 --
coau2I12 --
Gate1AA02 G1114 pAR0540 A1783 0 27 169 286 1065 4658 4715 4783 5764 12192
pAR1-03 --
pAR0503 pAR03H03 Gate3BF10 989 2016 4687 5372 10707
pGH742 P01-08 Gate1AF08 6117
Gate1CE09 --
Gate4CH11 pAR06G04 0 53 1028 1185 7112 10616
pAR0792 --
A1698 --
Gate2CC06 --
Unig22C05 --
pAR0248 0 846 8063 10578
A1706 A1216 0 59 615 11556
pAR0968 0
Unig23G01 0 11087
pVNC280 0 4928
P05-04 Coau2E05 BNL3860 P05-37 --
A1679 M16-088 A1731 P06-26 Gate1AF01 0
G1261 PVNC128 1304 2191
pAR0972 Gate2AC11 pAR0571 0 293 708 5608 6338 8905 9738 11158 11183 11733 11746 11924
Gafb16D09 --
Coau2A22 --
pVNC244 10383
pGH242 0 1131 3897 7820
Gate2BF03 0
Gate4DD02 --
BNL3556 --
pAR0571 0 5608 11733 11924
BNL3255 A1412 0 38 39 197 1768 6655 11496 11542 11587
G1074 Gate4AD12 9574
Gafb22M15 --
Gate4BG06 Unig22E12 BNL2961 Gate3AH02 0 27 127 290 2017 4468 10310 11255
pAR0813 0 2796 9724 10860 11395 11614 11711
Coau1O15 2318 12069
A1353 Gate4BE09 PXP1-77 0 5421
Unig06D12 0 3474 6309
pGH244 Gate4AD12 Unig22F09 0 27 159 3827
pGH724 --
A1108 --
AHD3 --
pAR0476 pGH797 --
Gate2CD09 PVNC035 --
Unig25B12 A1691 0 149 2100 5023 7720 10610 10633 10663 11132 11740 12052
pAR09A08 pAR0594 0 276 4928 11000 11565 12023
pAR0010 Gate1DE02 10744
Unig24D03 Coau1O15 286 2061 2318 3144 12069 12232
Gate1CE03 --
Unig27G11 A1562 pAR0785 pAR0523 0 9559 11620 12257
pGH317 0 10757
Coau1E19 --
Gate1CH03 --
Gate2BF02 pAR03A12 0 220 10899 11783
Gate4CF12 --
Gate3CE06 --
Gate2BC04 5394
Gate4DE09 pAR07D04 --
G1008 0 4355 6320
Gate1CF01 0 76 135 2163 7965
Unig24G08 Coau1G05 --
Gate3DD06 20
Unig22C03 0 106 152 1634 3038 4197 5424 6194 11753
pAR1-26 Gate3CH11 pAR07D04 pAR0218 0 989 10549 10642 12258
Gate4BG11 Gate3DE09 Gate1DF03 Gate2CC12 0 115 817 1436 3518 3808 11472
pVNC149 1304 2330 5804
pAR0854 pAR07D04 1653 6483 11395
pAR3-15 A1345 A1828 0 279 1785 2626 3043 3808 6306
BNL3792 pAR1-40 G1018 pAR0953 0 442 457 744 1094 4355 4520 4622 5792 6767 6847 9747 10931 11163 11391 11831
pAR06D11 --
Gate4AH08 Unig27F10 Coau2A11 0 20 1509 3138 11989
P10-56 --
Gate3DA02 Gate1AB06 Unig27F12 0 93 10732 11288 11299 12050
Coau2B08 pAR0118 pAR0798 Gate2CD01 P05-09 0 14 103 189 446 2330 2813 2843 4520 4981 5663 5804 6064 6214 7818 8073 9741 10736 10752 10
M16-200 A1667 Gate4AC08 pAR01D12 pGH318
Unig27C12 pVNC164 A1632 A1401 Gate4DA08 0 10799 11158 12140
pAR3-47 pAR0418
G1272 pAR04F07 0 7098 9865 10735 11117
pAR0118 A1590 0 2813 6064 9741 11154 11197
A1168 446
G1013 A1590 P05-24 Gate2CF02 0 17 2658 5493 12106
pGH422 --
BNL3257 P01-02 0 10591
pAR0973 --
Gate1CC03 0 20 11472
pAR0950 P05-18 pAR3-07 2016
Gate1DB08 pAR0332 0 283 763 2114 3589 10509 11293 12128
Gafb13B07 --
Unig06G04 0 481 1416 6309
Unig25C01 Gate1BF02 0 1108 4205
Gate3CH11 0 989 10549 10642 12258
Gate1BH09 --
Gate1AG10 pAR0309 Coau2C15 A1348 pAR4-11 0 282 11139 11435 12279 12281
G1010 0 228
Gate4DF01 --
Gate1CA10 340 9826
Coau2A22 --
pAR0789 --
BNL3627 --
pAR3-23 --
Coau1M06 AEST048 0 10451 10640
Gate1BF03 --
A1658 P01-46 A1197 100 3195 11685 11716
P07-04 --
pAR0978 0 183
Unig23H12 G1101 Gate2BC08 0 53 282 678 1263 1641 2776 3917 5804 10616 10745 10757 11139 12299
Gate3CG03 --
pAR01A05 --
pARC-04 --
Gate3BB05 G1078 Unig26G07 0 614 1340 2769
Gate4CB07 0
pAR0123 0
G1276 669
Gate3BE04 0 131 141 1990 10146 12054 12310
A1811 0 5179 9325 10679 10828 11275 11852
pAR0490 --
Gate4DG08 0 1641
Genetic map
(Homologous group 4)
Physical contigs

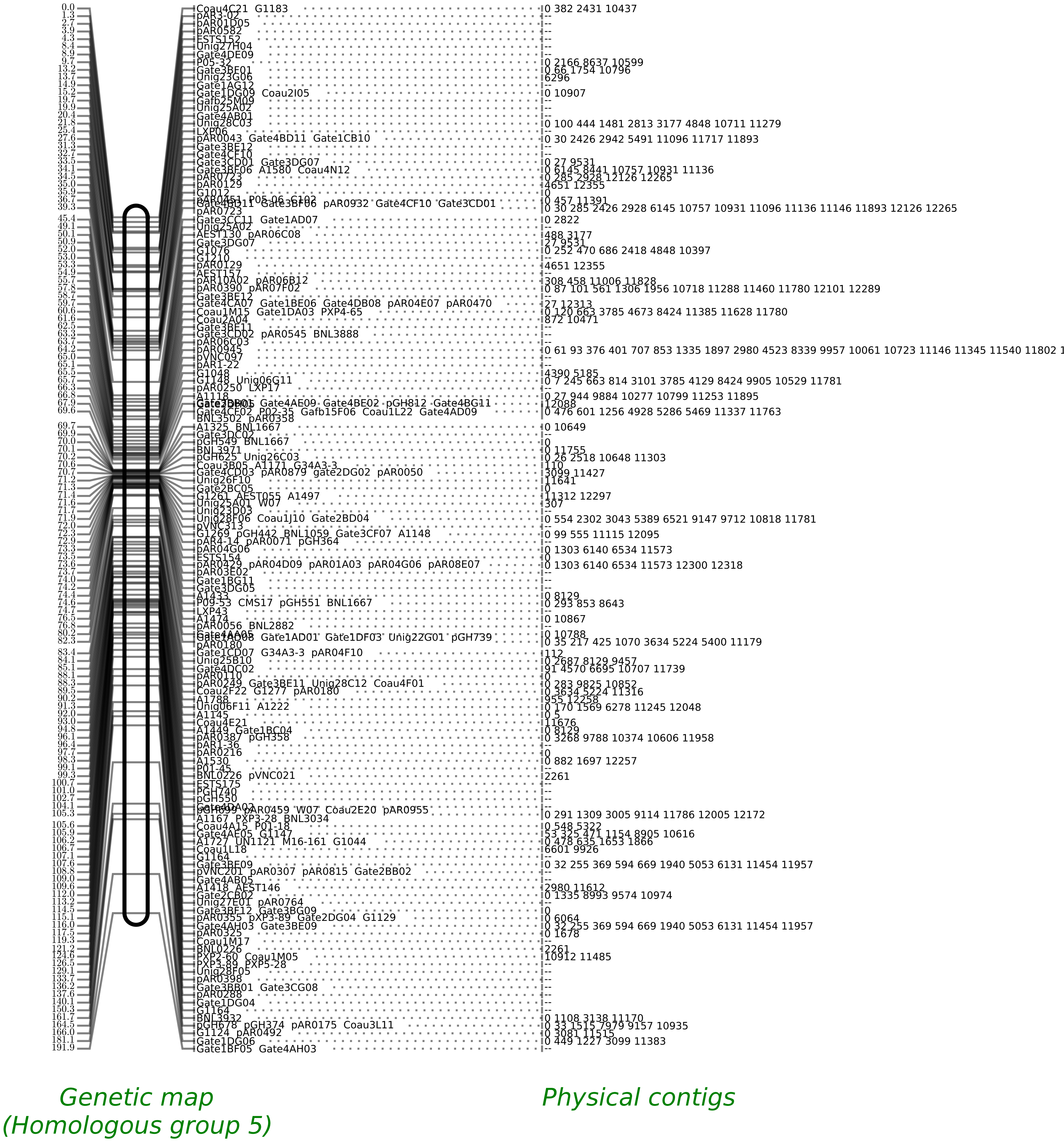
Genetic map
(Homologous group 5)
Physical contigs

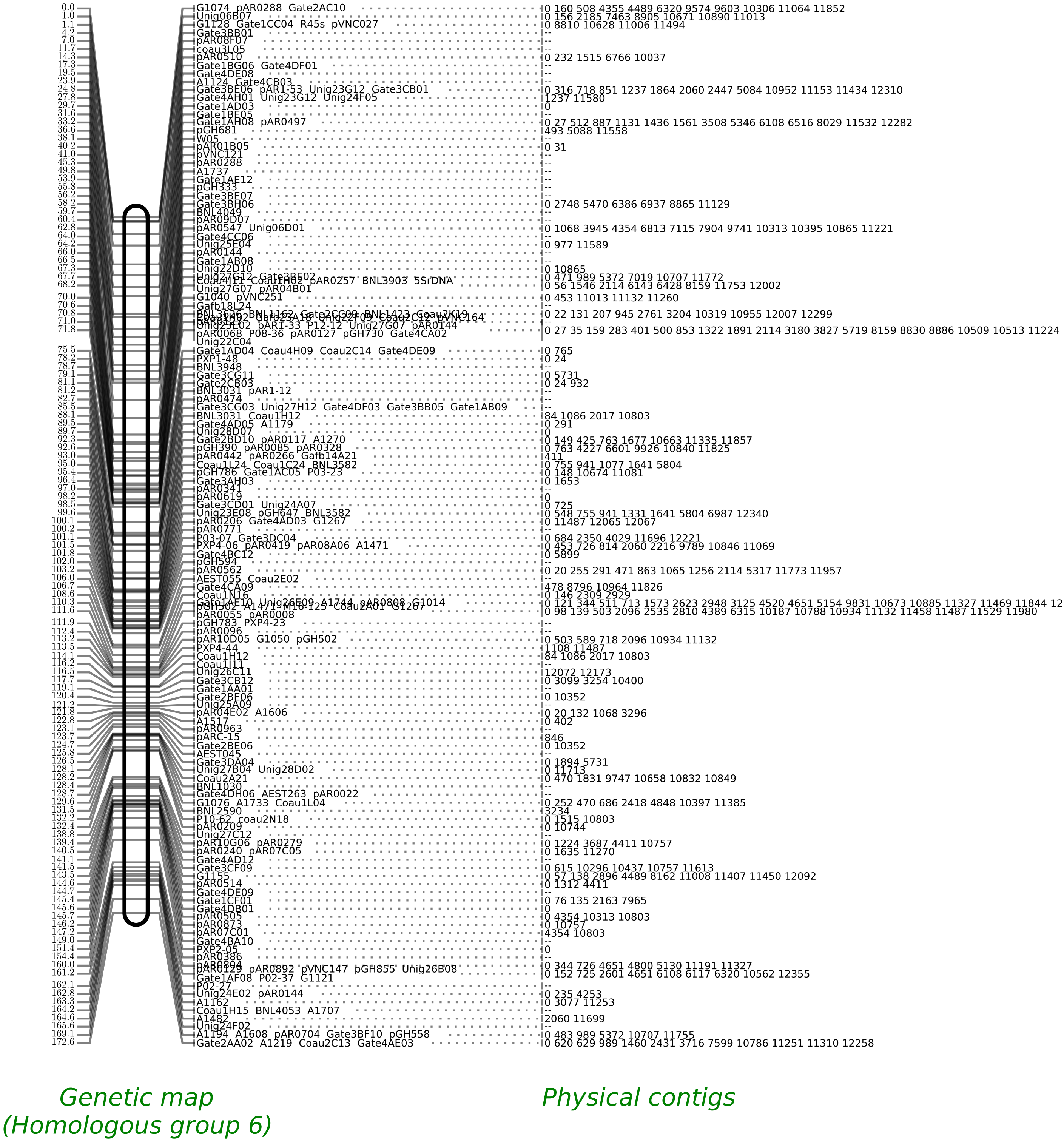
G1074 pAR0288 Gate2AC10
0 160 508 4355 4489 6320 9574 9603 10306 11064 11852
Unig06B07
0 156 2185 7463 8905 10671 10890 11013
G1128 Gate1CC04 R45s pVNC027
0 8810 10628 11006 11494
Gate3BB01
pAR08F07
coau3L05
pAR0510
0 232 1515 6766 10037
Gate1BG06 Gate4DF01
Gate4DE08
A1124 Gate4CB03
Gate3BE06 pAR1-53 Unig23G12 Gate3CB01
0 316 718 851 1237 1864 2060 2447 5084 10952 11153 11434 12310
Gate4AH01 Unig23G12 Unig24F05
1237 11580
Gate1AD03
Gate1BF05
Gate1AH08 pAR0497
0 27 512 887 1131 1436 1561 3508 5346 6108 6516 8029 11532 12282
pGH681
493 5088 11558
W05
pAR01B05
0 31
pVNC121
pAR0288
A1737
Gate1AE12
pGH333
Gate3BE07
Gate3BH06
0 2748 5470 6386 6937 8865 11129
BNL4049
pAR09D07
pAR0547 Unig06D01
0 1068 3945 4354 6813 7115 7904 9741 10313 10395 10865 11221
Gate4CC06
Unig25E04
0 977 11589
pAR0144
Gate1AB08
Unig22D10
0 10865
0 471 989 5372 7019 10707 11772
Coau4J11 Coau1H02 pAR0257 BNL3903 5SrDNA
0 56 1546 2114 6143 6428 8159 11753 12002
Unig27G07 pAR04B01
G1040 pVNC251
0 453 11013 11132 11260
Gafb18L24
0 22 131 207 945 2761 3204 10319 10955 12007 12299
Unig25E02 pAR1-33 P12-12 Unig27G07 pAR0144
pAR0068 P08-36 pAR0127 pGH730 Gate4CA02
0 27 35 159 283 401 500 853 1322 1891 2114 3180 3827 5719 8159 8830 8886 10509 10513 11224
Unig22C04
Gate1AD04 Coau4H09 Coau2C14 Gate4DE09
0 765
PXP1-48
0 24
BNL3948
Gate3CG11
0 5731
Gate2CB03
0 24 932
BNL3031 pAR1-12
pAR0474
Gate3CG03 Unig27H12 Gate4DF03 Gate3BB05 Gate1AB09
BNL3031 Coau1H12
84 1086 2017 10803
Gate4AD05 A1179
0 291
Unig28D07
Gate2BD10 pAR0117 A1270
0 149 425 763 1677 10663 11335 11857
pGH390 pAR0085 pAR0328
0 763 4227 6601 9926 10840 11825
pAR0442 pAR0266 Gafb14A21
411
Coau1L24 Coau1C24 BNL3582
0 755 941 1077 1641 5804
pGH786 Gate1AC05 P03-23
0 148 10674 11081
Gate3AH03
0 1653
pAR0341
pAR0619
Gate3CD01 Unig24A07
0 725
Unig23E08 pGH647 BNL3582
0 548 755 941 1331 1641 5804 6987 12340
pAR0206 Gate4AD03 G1267
0 11487 12065 12067
pAR0771
P03-07 Gate3DC04
0 684 2350 4029 11696 12221
PXP4-06 pAR0419 pAR08A06 A1471
0 453 726 814 2060 2216 9789 10846 11069
Gate4BC12
0 5899
pGH594
pAR0562
0 20 255 291 471 863 1065 1256 2114 5317 11773 11957
AEST055 Coau2E02
Gate4CA09
478 8796 10964 11826
Coau1N16
0 146 2309 2929
pAR0055 pAR0008
0 98 139 503 2096 2535 2810 4389 6315 10187 10788 10934 11132 11458 11487 11529 11980
pGH783 PXP4-23
pAR0096
pAR10D05 G1050 pGH502
0 503 589 718 2096 10934 11132
PXP4-44
1108 11487
Coau1H12
84 1086 2017 10803
Coau1J11
Unig26C11
12072 12173
Gate3CB12
0 3099 3254 10400
Gate1AA01
Gate2BE06
0 10352
Unig25A09
pAR04E02 A1606
0 20 132 1068 3296
A1517
0 402
pAR0963
pARC-15
846
Gate2BE06
0 10352
AEST045
Gate3DA04
0 1894 5731
Unig27B04 Unig28D02
0 11713
Coau2A21
0 470 1831 9747 10658 10832 10849
BNL1030
Gate4DH06 AEST263 pAR0022
G1076 A1733 Coau1L04
0 252 470 686 2418 4848 10397 11385
BNL2590
3234
P10-62 coau2N18
0 1515 10803
pAR0209
0 10744
Unig27C12
pAR10G06 pAR0279
0 1224 3687 4411 10757
pAR0240 pAR07C05
0 1635 11270
Gate4AD12
Gate3CF09
0 615 10296 10437 10757 11613
G1155
0 57 138 2896 4489 8162 11008 11407 11450 12092
pAR0514
0 1312 4411
Gate4DE09
Gate1CF01
0 76 135 2163 7965
Gate4DB01
pAR0505
0 4354 10313 10803
pAR0873
0 10757
pAR07C01
4354 10803
Gate4BA10
PXP2-05
pAR0386
0 344 726 4651 4800 5130 11191 11327
0 152 725 2601 4651 6108 6117 6320 10562 12355
Gate1AF08 P02-37 G1121
P02-27
Unig24E02 pAR0144
0 235 4253
A1162
0 3077 11253
Coau1H15 BNL4053 A1707
A1482
2060 11699
Unig24F02
A1194 A1608 pAR0704 Gate3BF10 pGH558
0 483 989 5372 10707 11755
Gate2AA02 A1219 Coau2C13 Gate4AE03
0 620 629 989 1460 2431 3716 7599 10786 11251 11310 12258
Genetic map
(Homologous group 6)
Physical contigs

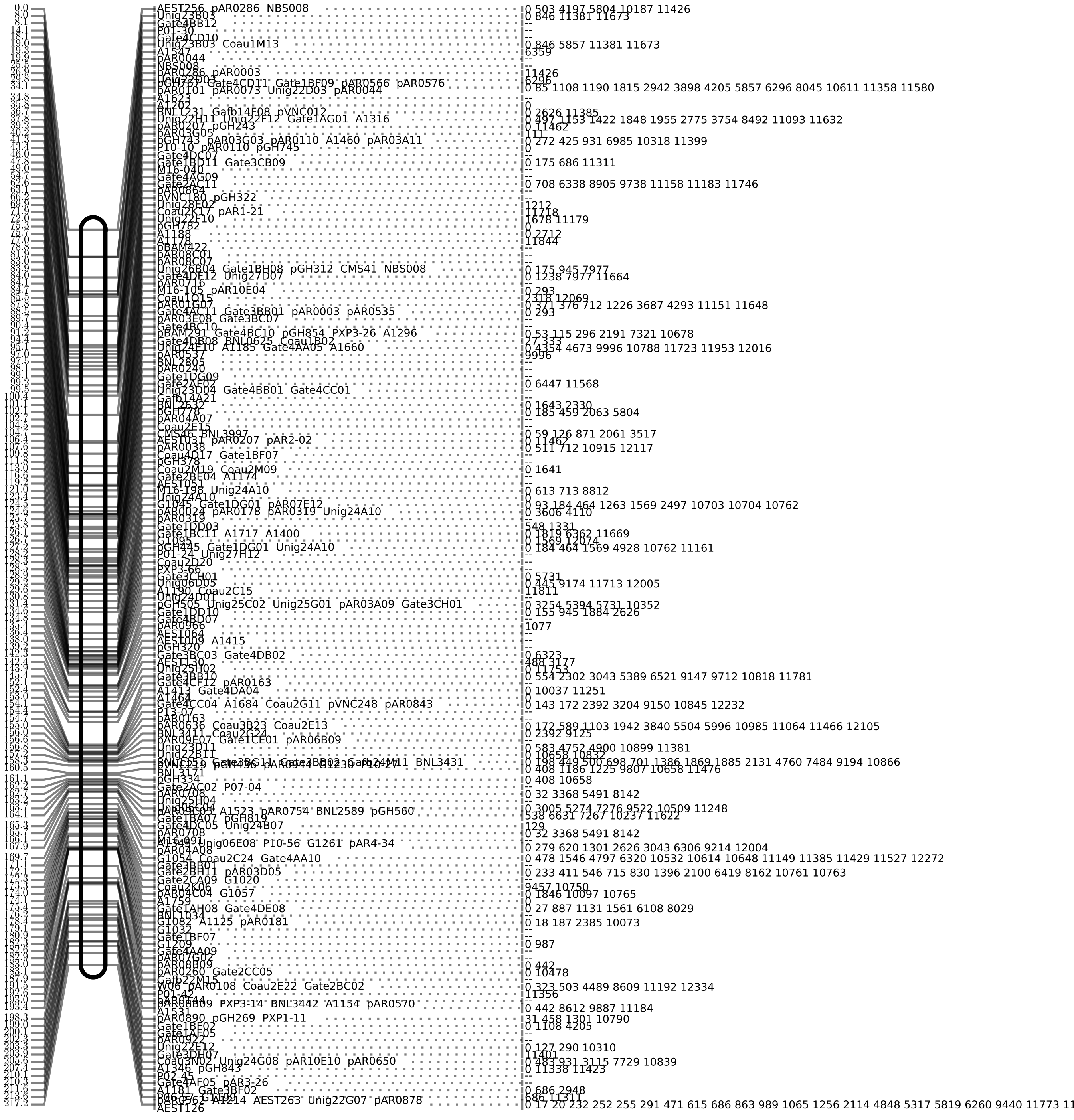

Genetic map
(Homologous group 7)

Physical contigs

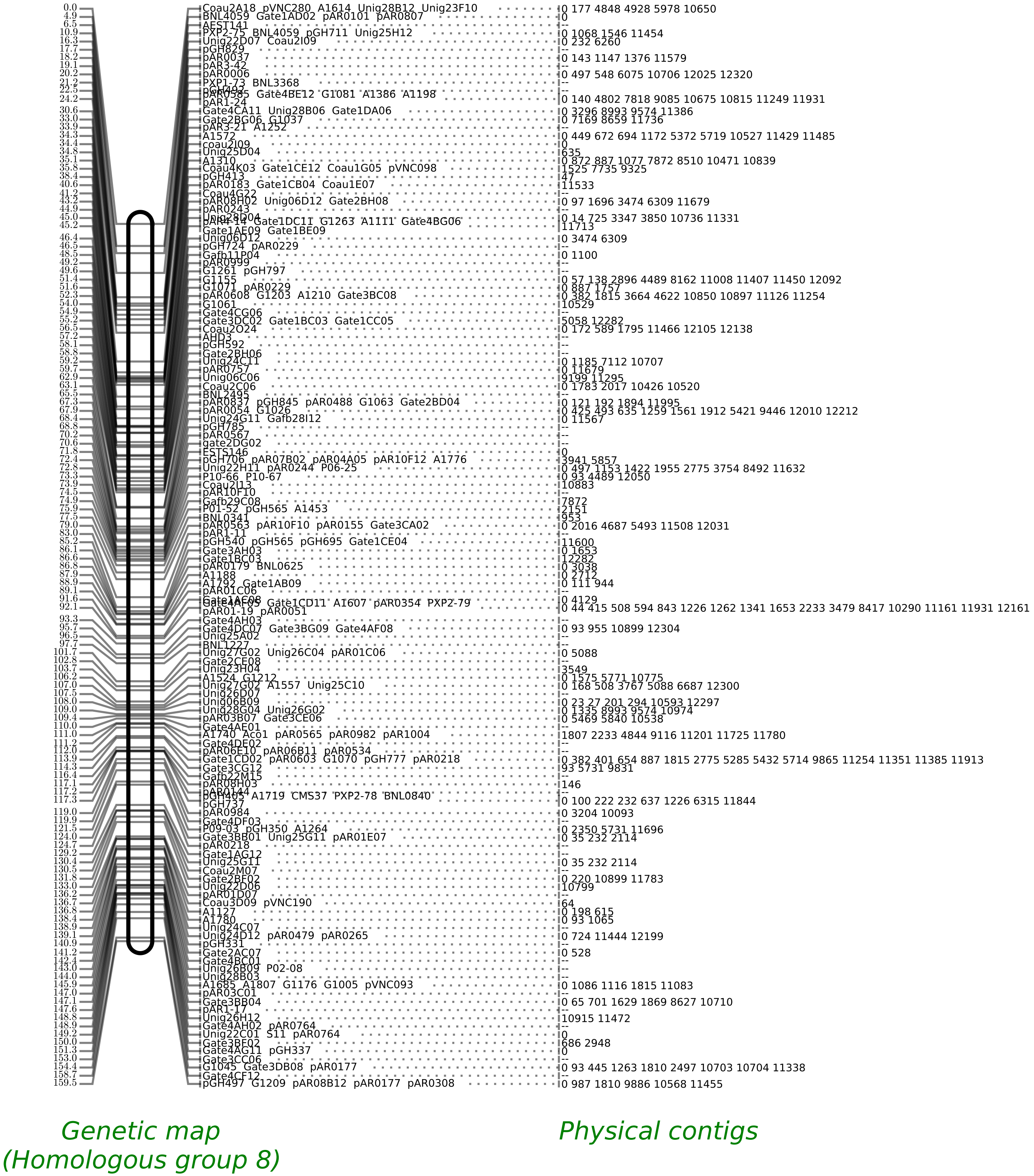
0.0 4.9 6.5 10.9 16.3 17.7 18.2 19.1 20.2 21.2 22.5 24.2 30.6 33.0 33.9 34.3 34.4 34.8 35.1 35.8 38.4 40.6 41.2 43.2 44.9 45.0 45.2 46.4 46.5 48.5 49.2 49.6 51.4 51.6 52.3 54.0 54.9 55.2 56.5 57.2 58.1 58.8 59.2 59.7 62.9 63.1 65.5 67.3 67.9 68.4 68.8 70.2 70.6 71.8 72.4 72.8 73.3 73.9 74.5 74.9 75.9 77.5 79.0 83.0 85.2 86.1 86.6 86.8 87.9 88.9 89.1 91.6 92.1 93.3 95.7 96.5 97.7 101.7 102.8 103.7 106.2 107.0 107.5 108.0 109.0 109.4 110.0 111.0 111.2 112.0 113.9 114.3 116.4 117.1 117.2 117.3 119.0 119.9 121.5 124.0 124.7 129.2 130.4 130.5 131.8 133.0 136.2 136.7 136.8 138.4 138.9 139.1 140.9 141.2 142.4 143.0 144.0 145.9 147.0 147.1 147.6 148.8 148.9 149.2 150.0 151.3 153.0 154.4 158.7 159.5
Coau2A18 pVNC280 A1614 Unig28B12 Unig23F10 — 0 177 4848 4928 5978 10650
BNL4059 Gate1AD02 pAR0101 pAR0807 — 0
AEST141 — --
PXP2-75 BNL4059 pGH711 Unig25H12 — 0 1068 1546 11454
Unig22D07 Coau2I09 — 0 232 6260
pGH829 — --
pAR0037 — 0 143 1147 1376 11579
pAR3-42 — --
pAR0006 — 0 497 548 6075 10706 12025 12320
PXP1-73 BNL3368 — --
pAR0585 Gate4BE12 G1081 A1386 A1198
pAR1-24 — 0 140 4802 7818 9085 10675 10815 11249 11931
Gate4CA11 Unig28B06 Gate1DA06 — 0 3296 8993 9574 11386
Gate2BG06 G1037 — 0 7169 8659 11736
pAR3-21 A1252 — --
A1572 — 0 449 672 694 1172 5372 5719 10527 11429 11485
coau2I09 — 0
Unig25D04 — 635
A1310 — 0 872 887 1077 7872 8510 10471 10839
Coau4K03 Gate1CE12 Coau1G05 pVNC098 — 1525 7735 9325
pGH413 — 47
pAR0183 Gate1CB04 Coau1E07 — 11533
Coau4G22 — --
pAR08H02 Unig06D12 Gate2BH08 — 0 97 1696 3474 6309 11679
pAR0243 — --
Unig28D04
pAR4-14 Gate1DC11 G1263 A1111 Gate4BG06 — 0 14 725 3347 3850 10736 11331
Gate1AE09 Gate1BE09 — 11713
Unig06D12 — 0 3474 6309
pGH724 pAR0229 — --
Gafb11P04 — 0 1100
pAR0999 — --
G1261 pGH797 — --
G1155 — 0 57 138 2896 4489 8162 11008 11407 11450 12092
G1071 pAR0229 — 0 887 1757
pAR0608 G1203 A1210 Gate3BC08 — 0 382 1815 3664 4622 10850 10897 11126 11254
G1061 — 10529
Gate4CG06 — --
Gate3DC02 Gate1BC03 Gate1CC05 — 5058 12282
Coau2O24 — 0 172 589 1795 11466 12105 12138
AHD3 — --
pGH592 — --
Gate2BH06 — --
Unig24C11 — 0 1185 7112 10707
pAR0757 — 0 11679
Unig06C06 — 9199 11295
Coau2C06 — 0 1783 2017 10426 10520
BNL2495 — --
pAR0837 pGH845 pAR0488 G1063 Gate2BD04 — 0 121 192 1894 11995
pAR0054 G1026 — 0 425 493 635 1259 1561 1912 5421 9446 12010 12212
Unig24G11 Gafb28I12 — 0 11567
pGH785 — --
pAR0567 — --
gate2DG02 — --
ESTS146 — 0
pGH706 pAR07B02 pAR04A05 pAR10F12 A1776 — 3941 5857
Unig22H11 pAR0244 P06-25 — 0 497 1153 1422 1955 2775 3754 8492 11632
P10-66 P10-67 — 0 93 4489 12050
Coau2I13 — 10883
pAR10F10 — --
Gafb29C08 — 7872
P01-52 pGH565 A1453 — 2151
BNL0341 — 953
pAR0563 pAR10F10 pAR0155 Gate3CA02 — 0 2016 4687 5493 11508 12031
pAR1-11 — --
pGH540 pGH565 pGH695 Gate1CE04 — 11600
Gate3AH03 — 0 1653
Gate1BC03 — 12282
pAR0179 BNL0625 — 0 3038
A1188 — 0 2712
A1792 Gate1AB09 — 0 111 944
pAR01C06 — --
Gate4AF05 Gate1CD11 A1607 pAR0354 PXP2-79 — 0 4129
pAR01-19 pAR0051 — 0 44 415 508 594 843 1226 1262 1341 1653 2233 3479 8417 10290 11161 11931 12161
Gate4AH03 — --
Gate4DC07 Gate3BG09 Gate4AF08 — 0 93 955 10899 12304
Unig25A02 — --
BNL1227 — --
Unig27G02 Unig26C04 pAR01C06 — 0 5088
Gate2CE08 — --
Unig23H04 — 3549
A1524 G1212 — 0 1575 5771 10775
Unig27G02 A1557 Unig25C10 — 0 168 508 3767 5088 6687 12300
Unig26D07 — --
Unig06B09 — 0 23 27 201 294 10593 12297
Unig28G04 Unig26G02 — 0 1335 8993 9574 10974
pAR03B07 Gate3CE06 — 0 5469 5840 10538
Gate4AE01 — --
A1740 Aco1 pAR0565 pAR0982 pAR1004 — 1807 2233 4844 9116 11201 11725 11780
Gate4DE02 — --
pAR06E10 pAR06B11 pAR0534 — --
Gate1CD02 pAR0603 G1070 pGH777 pAR0218 — 0 382 401 654 887 1815 2775 5285 5432 5714 9865 11254 11351 11385 11913
Gate3CG12 — 93 5731 9831
Gafb22M15 — --
pAR08H03 — 146
pAR0144 — --
pGH405 A1719 CMS37 PXP2-78 BNL0840 — 0 100 222 232 637 1226 6315 11844
pGH737
pAR0984 — 0 3204 10093
Gate4DF03 — --
P09-03 pGH350 A1264 — 0 2350 5731 11696
Gate3BB01 Unig25G11 pAR01E07 — 0 35 232 2114
pAR0218 — --
Gate1AG12 — --
Unig25G11 — 0 35 232 2114
Coau2M07 — --
Gate2BF02 — 0 220 10899 11783
Unig22D06 — 10799
pAR01D07 — --
Coau3D09 pVNC190 — 64
A1127 — 0 198 615
A1780 — 0 93 1065
Unig24C07 — --
Unig24D12 pAR0479 pAR0265 — 0 724 11444 12199
pGH331 — --
Gate2AC07 — 0 528
Gate4BC01 — --
Unig26B09 P02-08 — --
Unig28B03 — --
A1685 A1807 G1176 G1005 pVNC093 — 0 1086 1116 1815 11083
pAR03C01 — --
Gate3BB04 — 0 65 701 1629 1869 8627 10710
pAR1-17 — --
Unig26H12 — 10915 11472
Gate4AH02 pAR0764 — --
Unig22C01 S11 pAR0764 — 0
Gate3BF02 — 686 2948
Gate4AG11 pGH337 — 0
Gate3CC06 — --
G1045 Gate3DB08 pAR0177 — 0 93 445 1263 1810 2497 10703 10704 11338
Gate4CF12 — --
pGH497 G1209 pAR08B12 pAR0177 pAR0308 — 0 987 1810 9886 10568 11455
Genetic map
(Homologous group 8)
Physical contigs

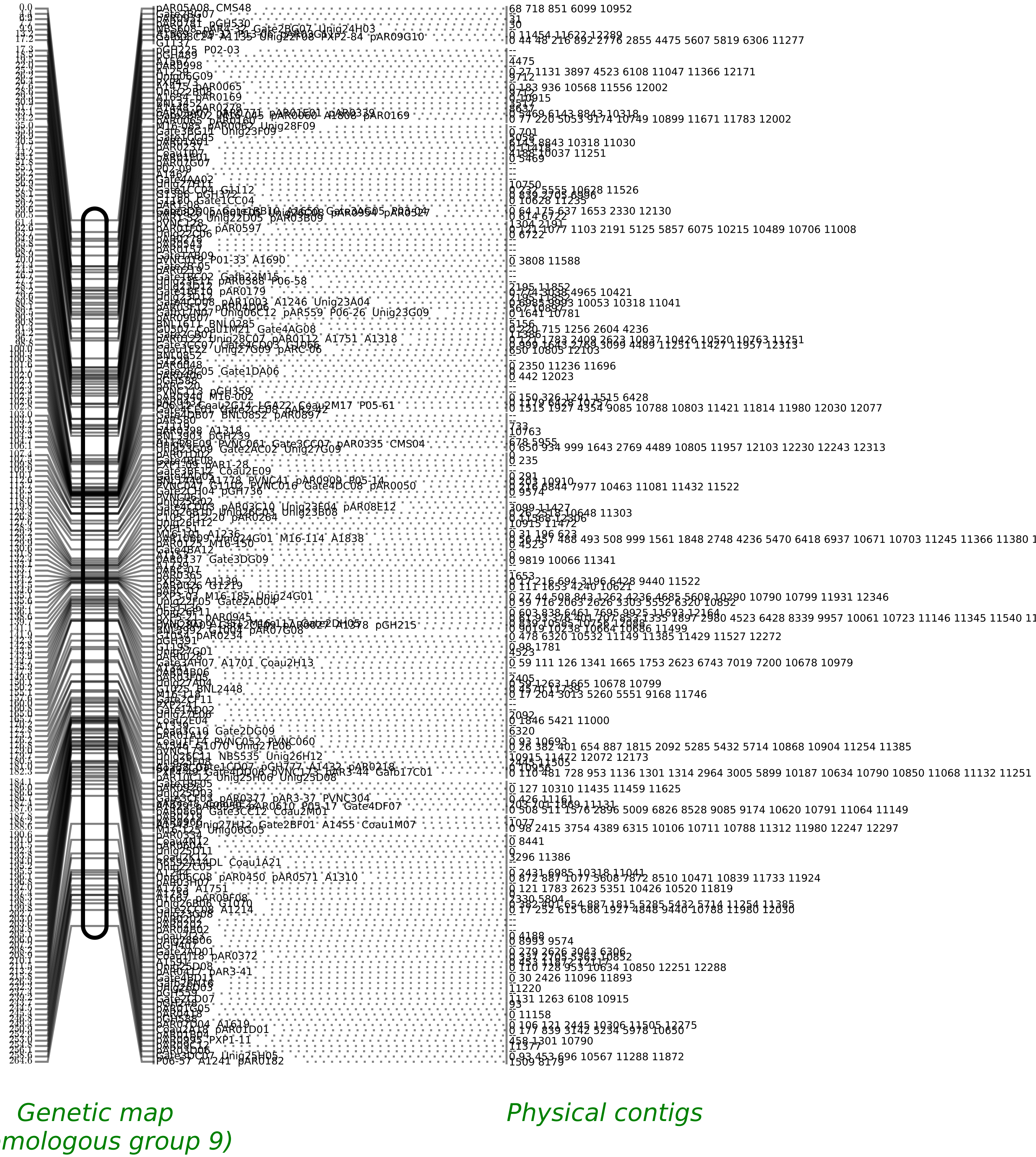
Genetic map
(Homologous group 9)
Physical contigs

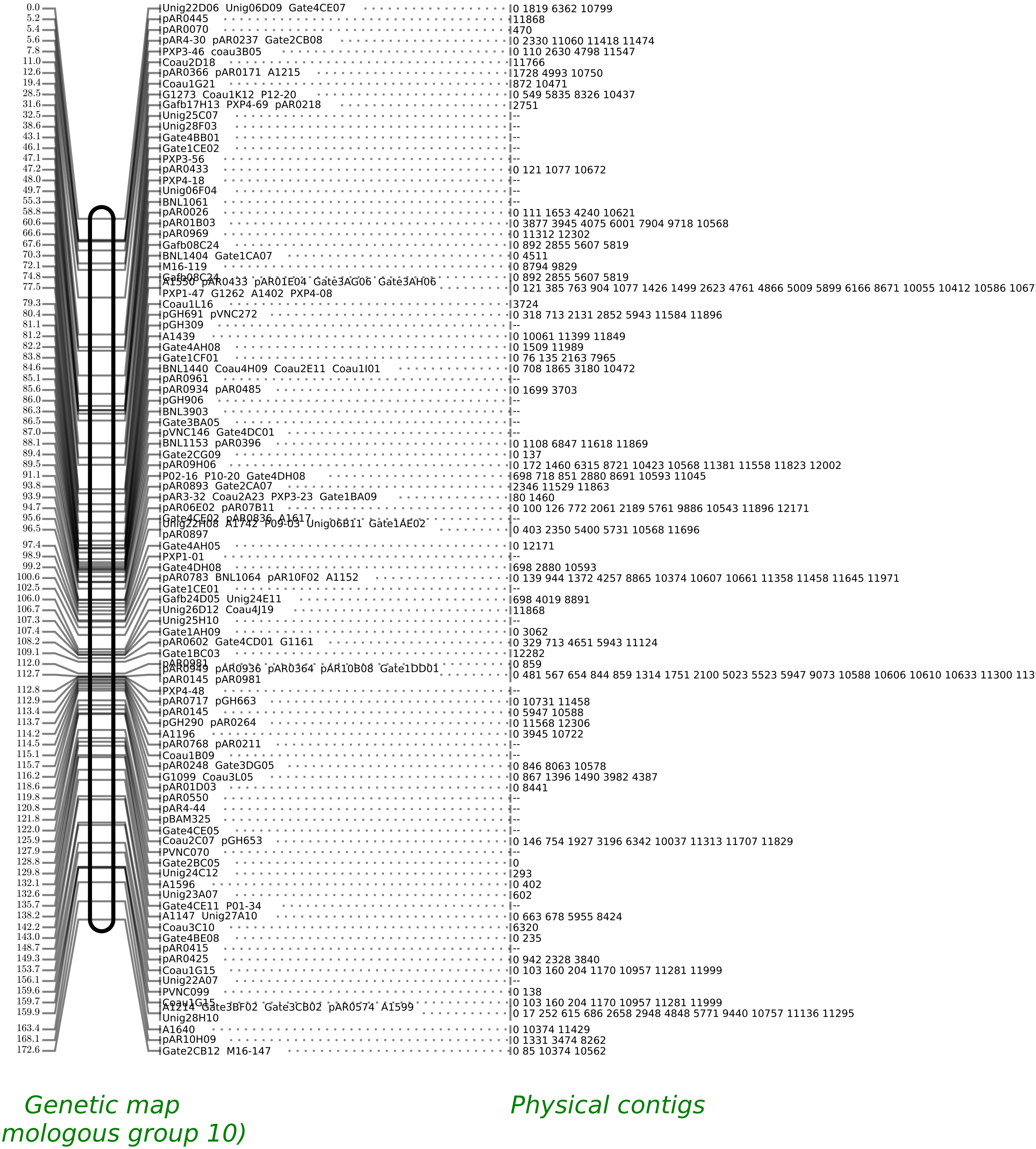

0.0
5.2
5.4
5.6
7.8
11.0
12.6
19.4
28.5
31.6
32.5
38.6
43.1
46.1
47.1
47.2
48.0
49.7
55.3
58.8
60.6
66.6
67.6
70.3
72.1
74.8
77.5
79.3
80.4
81.1
81.2
82.2
83.8
84.6
85.1
85.6
86.0
86.3
86.5
87.0
88.1
89.4
89.5
91.1
93.8
93.9
94.7
95.6
96.5
97.4
98.9
99.2
100.6
102.5
106.0
106.7
107.3
107.4
108.2
109.1
112.0
112.7
112.8
112.9
113.4
113.7
114.2
114.5
115.1
115.7
116.2
118.6
119.8
120.8
121.8
122.0
125.9
127.9
128.8
129.8
132.1
132.6
135.7
138.2
142.2
143.0
148.7
149.3
153.7
156.1
159.6
159.7
159.9
163.4
168.1
172.6
Unig22D06 Unig06D09 Gate4CE07
pAR0445
pAR0070
pAR4-30 pAR0237 Gate2CB08
PXP3-46 coau3B05
Coau2D18
pAR0366 pAR0171 A1215
Coau1G21
G1273 Coau1K12 P12-20
Gafb17H13 PXP4-69 pAR0218
Unig25C07
Unig28F03
Gate4BB01
Gate1CE02
PXP3-56
pAR0433
PXP4-18
Unig06F04
BNL1061
pAR0026
pAR01B03
pAR0969
Gafb08C24
BNL1404 Gate1CA07
M16-119
Gafb08C24
A1550 pAR0433 pAR01E04 Gate3AG06 Gate3AH06
PXP1-47 G1262 A1402 PXP4-08
Coau1L16
pGH691 pVNC272
pGH309
A1439
Gate4AH08
Gate1CF01
BNL1440 Coau4H09 Coau2E11 Coau1I01
pAR0961
pAR0934 pAR0485
pGH906
BNL3903
Gate3BA05
pVNC146 Gate4DC01
BNL1153 pAR0396
Gate2CG09
pAR09H06
P02-16 P10-20 Gate4DH08
pAR0893 Gate2CA07
pAR3-32 Coau2A23 PXP3-23 Gate1BA09
pAR06E02 pAR07B11
Gate4CE02 pAR0836 A1617
Unig22H08 A1742 P09-03 Unig06B11 Gate1AE02
pAR0897
Gate4AH05
PXP1-01
Gate4DH08
pAR0783 BNL1064 pAR10F02 A1152
Gate1CE01
Gafb24D05 Unig24E11
Unig26D12 Coau4J19
Unig25H10
Gate1AH09
pAR0602 Gate4CD01 G1161
Gate1BC03
pAR0981
pAR0949 pAR0936 pAR0364 pAR10B08 Gate1DD01
pAR0145 pAR0981
PXP4-48
pAR0717 pGH663
pAR0145
pGH290 pAR0264
A1196
pAR0768 pAR0211
Coau1B09
pAR0248 Gate3DG05
G1099 Coau3L05
pAR01D03
pAR0550
pAR4-44
pBAM325
Gate4CE05
Coau2C07 pGH653
PVNC070
Gate2BC05
Unig24C12
A1596
Unig23A07
Gate4CE11 P01-34
A1147 Unig27A10
Coau3C10
Gate4BE08
pAR0415
pAR0425
Coau1G15
Unig22A07
PVNC099
Coau1G15
A1214 Gate3BF02 Gate3CB02 pAR0574 A1599
Unig28H10
A1640
pAR10H09
Gate2CB12 M16-147
0 1819 6362 10799
11868
470
0 2330 11060 11418 11474
0 110 2630 4798 11547
11766
1728 4993 10750
872 10471
0 549 5835 8326 10437
2751
--
--
--
--
--
0 121 1077 10672
--
--
--
0 111 1653 4240 10621
0 3877 3945 4075 6001 7904 9718 10568
0 11312 12302
0 892 2855 5607 5819
0 4511
0 8794 9829
0 892 2855 5607 5819
0 121 385 763 904 1077 1426 1499 2623 4761 4866 5009 5899 6166 8671 10055 10412 10586 1067
3724
0 318 713 2131 2852 5943 11584 11896
--
0 10061 11399 11849
0 1509 11989
0 76 135 2163 7965
0 708 1865 3180 10472
--
0 1699 3703
--
--
--
--
0 1108 6847 11618 11869
0 137
0 172 1460 6315 8721 10423 10568 11381 11558 11823 12002
698 718 851 2880 8691 10593 11045
2346 11529 11863
80 1460
0 100 126 772 2061 2189 5761 9886 10543 11896 12171
--
0 403 2350 5400 5731 10568 11696
0 12171
--
698 2880 10593
0 139 944 1372 4257 8865 10374 10607 10661 11358 11458 11645 11971
--
698 4019 8891
11868
--
0 3062
0 329 713 4651 5943 11124
12282
0 859
0 481 567 654 844 859 1314 1751 2100 5023 5523 5947 9073 10588 10606 10610 10633 11300 113
--
0 10731 11458
0 5947 10588
0 11568 12306
0 3945 10722
--
--
0 846 8063 10578
0 867 1396 1490 3982 4387
0 8441
--
--
--
--
0 146 754 1927 3196 6342 10037 11313 11707 11829
--
0
293
0 402
602
--
0 663 678 5955 8424
6320
0 235
--
0 942 2328 3840
0 103 160 204 1170 10957 11281 11999
--
0 138
0 103 160 204 1170 10957 11281 11999
0 17 252 615 686 2658 2948 4848 5771 9440 10757 11136 11295
0 10374 11429
0 1331 3474 8262
0 85 10374 10562
Genetic map
(Homologous group 10)
Physical contigs

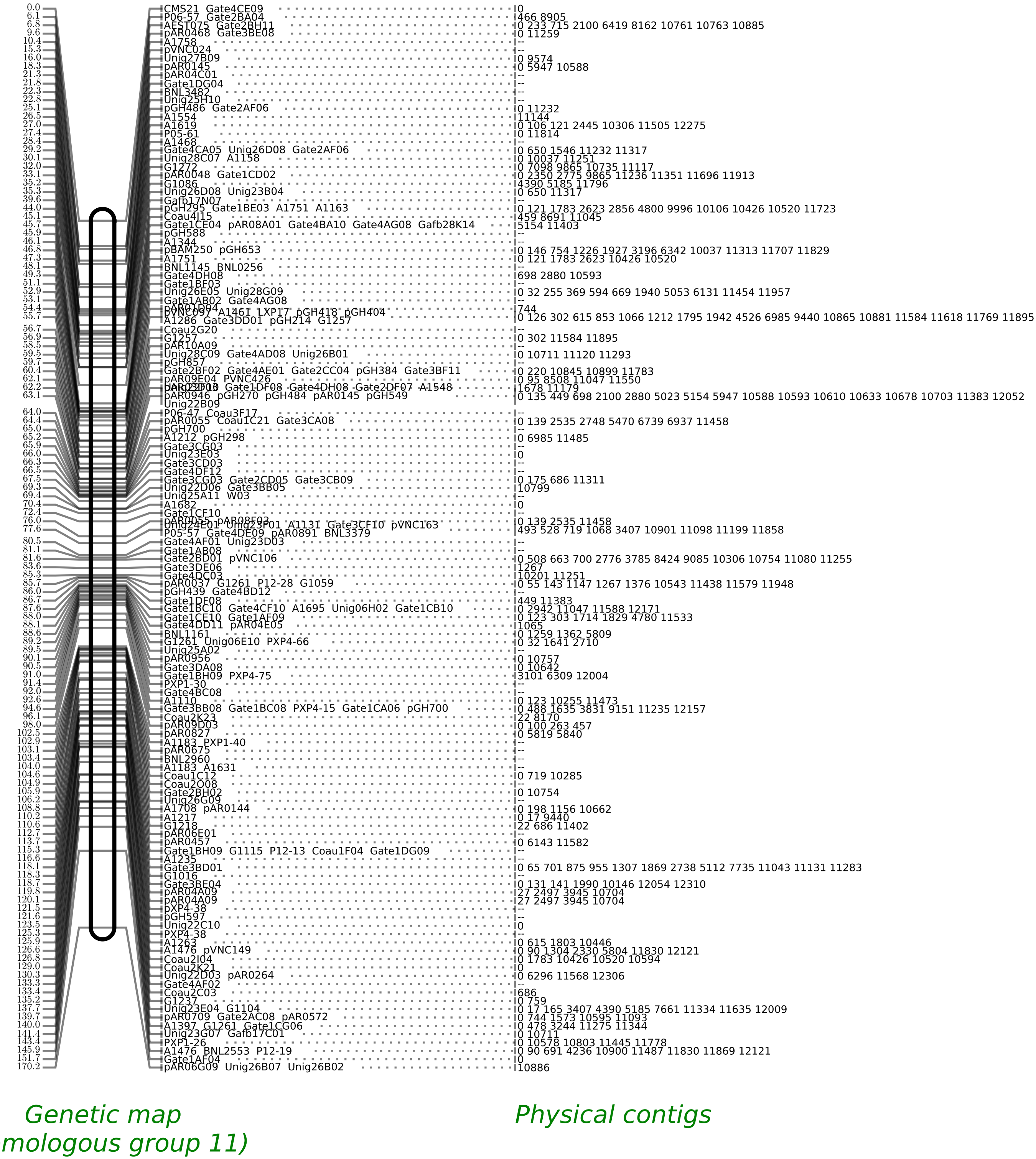

Genetic map
(Homologous group 11)
Physical contigs

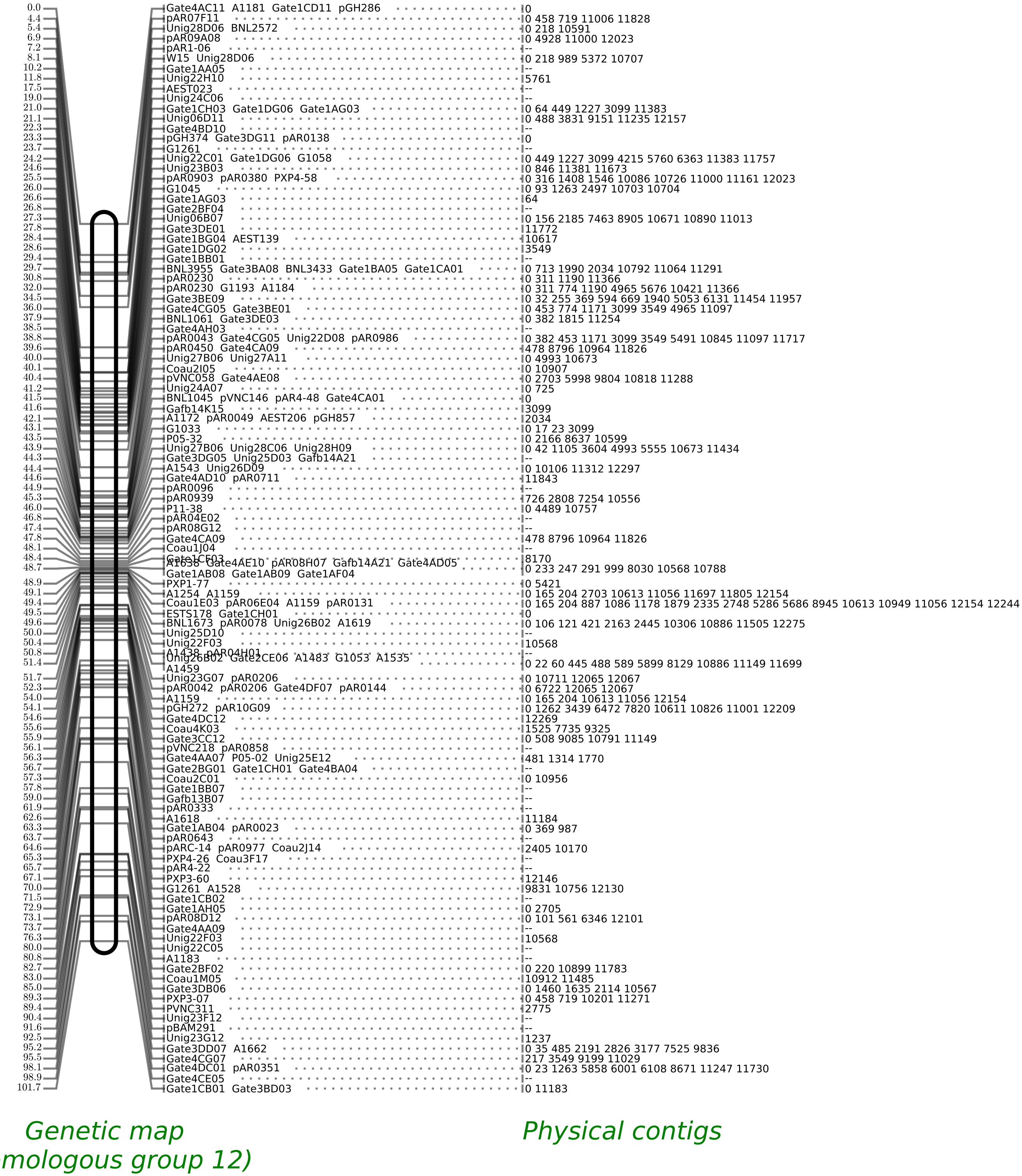

0.0 Gate4AC11 A1181 Gate1CD11 pGH286 0
4.4 pAR07F11 0 458 719 11006 11828
5.4 Unig28D06 BNL2572 0 218 10591
6.9 pAR09A08 0 4928 11000 12023
7.2 pAR1-06 --
8.1 W15 Unig28D06 0 218 989 5372 10707
10.2 Gate1AA05 --
11.8 Unig22H10 5761
17.5 AEST023 --
19.0 Unig24C06 --
21.0 Gate1CH03 Gate1DG06 Gate1AG03 0 64 449 1227 3099 11383
21.1 Unig06D11 0 488 3831 9151 11235 12157
22.3 Gate4BD10 --
23.3 pGH374 Gate3DG11 pAR0138 0
23.7 G1261 --
24.2 Unig22C01 Gate1DG06 G1058 0 449 1227 3099 4215 5760 6363 11383 11757
24.6 Unig23B03 0 846 11381 11673
25.5 pAR0903 pAR0380 PXP4-58 0 316 1408 1546 10086 10726 11000 11161 12023
26.0 G1045 0 93 1263 2497 10703 10704
26.6 Gate1AG03 64
26.8 Gate2BF04 --
27.3 Unig06B07 0 156 2185 7463 8905 10671 10890 11013
27.8 Gate3DE01 11772
28.4 Gate1BG04 AEST139 10617
28.6 Gate1DG02 3549
29.4 Gate1BB01 --
29.7 BNL3955 Gate3BA08 BNL3433 Gate1BA05 Gate1CA01 0 713 1990 2034 10792 11064 11291
30.8 pAR0230 0 311 1190 11366
32.0 pAR0230 G1193 A1184 0 311 774 1190 4965 5676 10421 11366
34.5 Gate3BE09 0 32 255 369 594 669 1940 5053 6131 11454 11957
36.0 Gate4CG05 Gate3BE01 0 453 774 1171 3099 3549 4965 11097
37.9 BNL1061 Gate3DE03 0 382 1815 11254
38.5 Gate4AH03 --
38.8 pAR0043 Gate4CG05 Unig22D08 pAR0986 0 382 453 1171 3099 3549 5491 10845 11097 11717
39.6 pAR0450 Gate4CA09 478 8796 10964 11826
40.0 Unig27B06 Unig27A11 0 4993 10673
40.1 Coau2I05 0 10907
40.4 pVNC058 Gate4AE08 0 2703 5998 9804 10818 11288
41.2 Unig24A07 0 725
41.5 BNL1045 pVNC146 pAR4-48 Gate4CA01 0
41.6 Gafb14K15 3099
42.1 A1172 pAR0049 AEST206 pGH857 2034
43.1 G1033 0 17 23 3099
43.5 P05-32 0 2166 8637 10599
43.9 Unig27B06 Unig28C06 Unig28H09 0 42 1105 3604 4993 5555 10673 11434
44.3 Gate3DG05 Unig25D03 Gafb14A21 --
44.4 A1543 Unig26D09 0 10106 11312 12297
44.6 Gate4AD10 pAR0711 11843
44.9 pAR0096 --
45.3 pAR0939 726 2808 7254 10556
46.0 P11-38 0 4489 10757
46.8 pAR04E02 --
47.4 pAR08G12 --
47.8 Gate4CA09 478 8796 10964 11826
48.1 Coau1J04 --
48.4 Gate1CF03 8170
48.7 A1668 Gate4AE10 pAR08H07 Gafb14A21 Gate4AD05 Gate1AB08 Gate1AB09 Gate1AF04 0 233 247 291 999 8030 10568 10788
48.9 PXP1-77 0 5421
49.1 A1254 A1159 0 165 204 2703 10613 11056 11697 11805 12154
49.4 Coau1E03 pAR06E04 A1159 pAR0131 0 165 204 887 1086 1178 1879 2335 2748 5286 5686 8945 10613 10949 11056 12154 12244
49.5 ESTS178 Gate1CH01 0
49.6 BNL1673 pAR0078 Unig26B02 A1619 0 106 121 421 2163 2445 10306 10886 11505 12275
50.0 Unig25D10 --
50.4 Unig22F03 10568
50.8 A1438 pAR04H01 --
51.4 Unig26B02 Gate2CE06 A1483 G1053 A1535 A1459 0 22 60 445 488 589 5899 8129 10886 11149 11699
51.7 Unig23G07 pAR0206 0 10711 12065 12067
52.3 pAR0042 pAR0206 Gate4DF07 pAR0144 0 6722 12065 12067
54.0 A1159 0 165 204 10613 11056 12154
54.1 pGH272 pAR10G09 0 1262 3439 6472 7820 10611 10826 11001 12209
54.6 Gate4DC12 12269
55.6 Coau4K03 1525 7735 9325
55.9 Gate3CC12 0 508 9085 10791 11149
56.1 pVNC218 pAR0858 --
56.3 Gate4AA07 P05-02 Unig25E12 481 1314 1770
56.7 Gate2BG01 Gate1CH01 Gate4BA04 --
57.3 Coau2C01 0 10956
57.8 Gate1BB07 --
59.0 Gafb13B07 --
61.9 pAR0333 --
62.6 A1618 11184
63.3 Gate1AB04 pAR0023 0 369 987
63.7 pAR0643 --
64.6 pARC-14 pAR0977 Coau2J14 2405 10170
65.3 PXP4-26 Coau3F17 --
65.7 pAR4-22 --
67.1 PXP3-60 12146
70.0 G1261 A1528 9831 10756 12130
71.5 Gate1CB02 --
72.9 Gate1AH05 0 2705
73.1 pAR08D12 0 101 561 6346 12101
73.7 Gate4AA09 --
76.3 Unig22F03 10568
80.0 Unig22C05 --
80.8 A1183 --
82.7 Gate2BF02 0 220 10899 11783
83.0 Coau1M05 10912 11485
85.0 Gate3DB06 0 1460 1635 2114 10567
89.3 PXP3-07 0 458 719 10201 11271
89.4 PVNC311 2775
90.4 Unig23F12 --
91.6 pBAM291 --
92.5 Unig23G12 1237
95.2 Gate3DD07 A1662 0 35 485 2191 2826 3177 7525 9836
95.5 Gate4CG07 217 3549 9199 11029
98.1 Gate4DC01 pAR0351 0 23 1263 5858 6001 6108 8671 11247 11730
98.9 Gate4CE05 --
101.7 Gate1CB01 Gate3BD03 0 11183
Genetic map
(Homologous group 12)
Physical contigs

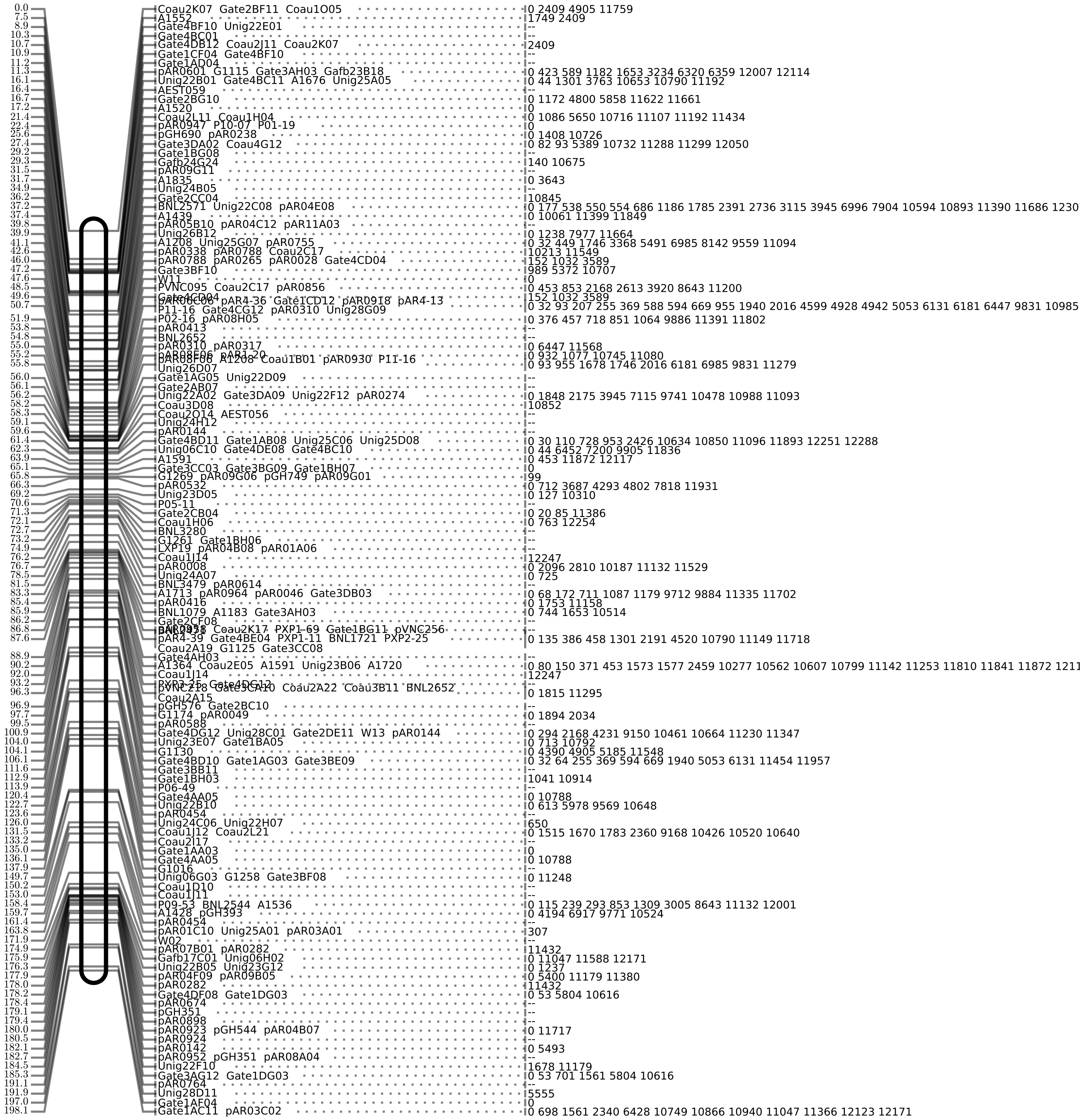

Genetic map (Homologous group 13)

Physical contigs
