# Supplementary material for: A draft physical map of a D-genome cotton species (Gossypium raimondii)
Source: BMC Genomics. 2010 Jun 22;11:395. doi: 10.1186/1471-2164-11-395 (PMC2996926; doi:10.1186/1471-2164-11-395)
Supplement: Additional file 6 — G. raimondii preliminary gene ontology classification results. GO classification results generated from 13662 BAC-end sequences and 13661 random shotgun sequences, using Blast2Go at an ontology level of 2. [file 1471-2164-11-395-S6.DOC]

TABLE S1: GO classification results generated from 13662 BAC-end sequences and 13661 random shotgun sequences, using Blast2Go at an ontology level of 2

|  | BES |  | random shotgun |  |
| --- | --- | --- | --- | --- |
| reproduction | 7 | 0.15% | 7 | 0.18% |
| reproductive process | 7 | 0.15% | 7 | 0.18% |
| multicellular organismal process | 4 | 0.09% | 13 | 0.34% |
| viral reproduction | 1 | 0.02% | 1 | 0.03% |
| establishment of localization | 87 | 1.86% | 148 | 3.87% |
| immune system process | 4 | 0.09% | 2 | 0.05% |
| metabolic process | 2034 | 43.59% | 1585 | 41.44% |
| biological regulation | 92 | 1.97% | 88 | 2.30% |
| response to stimulus | 60 | 1.29% | 58 | 1.52% |
| cellular process | 2094 | 44.88% | 1604 | 41.93% |
| locomotion | 1 | 0.02% | - | - |
| biological adhesion | - | - | 1 | 0.03% |
| growth | - | - | 1 | 0.03% |
| localization | 88 | 1.89% | 148 | 3.87% |
| developmental process | 177 | 3.79% | 155 | 4.05% |
| multi-organism process | 10 | 0.21% | 7 | 0.18% |
| Total | 4666 |  | 3825 |  |
